# Gestationally dependent immune organization at the maternal-fetal interface

**DOI:** 10.1016/j.celrep.2022.111651

**Published:** 2022-11-15

**Authors:** Amber R. Moore, Nora Vivanco Gonzalez, Katherine A. Plummer, Olivia R. Mitchel, Harleen Kaur, Moises Rivera, Brian Collica, Mako Goldston, Ferda Filiz, Michael Angelo, Theo D. Palmer, Sean C. Bendall

**Affiliations:** 1 Immunology Graduate Program, Stanford University, Stanford, CA 94305, USA; 2 Department of Pathology, Stanford University, Stanford, CA 94305, USA; 3 Department of Neurosurgery, Institute for Stem Cell Biology and Regenerative Medicine, Stanford University, Stanford, CA 94305, USA

**Keywords:** maternal-fetal interface, placenta, mass cytometry, CyTOF, myeloid diversity, neutrophils, mononuclear phagocytes, PD-L1, heterogeneity, tissue specialization, microenvironment

## Abstract

The immune system and placenta have a dynamic relationship across gestation to accommodate fetal growth and development. High-resolution characterization of this maternal-fetal interface is necessary to better understand the immunology of pregnancy and its complications. We developed a single-cell framework to simultaneously immuno-phenotype circulating, endovascular, and tissue-resident cells at the maternal-fetal interface throughout gestation, discriminating maternal and fetal contributions. Our data reveal distinct immune profiles across the endovascular and tissue compartments with tractable dynamics throughout gestation that respond to a systemic immune challenge in a gestationally dependent manner. We uncover a significant role for the innate immune system where phagocytes and neutrophils drive temporal organization of the placenta through remarkably diverse populations, including PD-L1^+^ subsets having compartmental and early gestational bias. Our approach and accompanying datasets provide a resource for additional investigations into gestational immunology and evoke a more significant role for the innate immune system in establishing the microenvironment of early pregnancy.

## Highlights

• A framework to assess perturbations of immune homeostasis in pregnancy
• Endovascular compartment imparts immune regulation at the maternal-fetal interface
• Identification of pregnancy-specific immune network and coordination
• Phenotypic specialization of innate cells to placental microenvironments

## Graphical abstract

**Figure:**
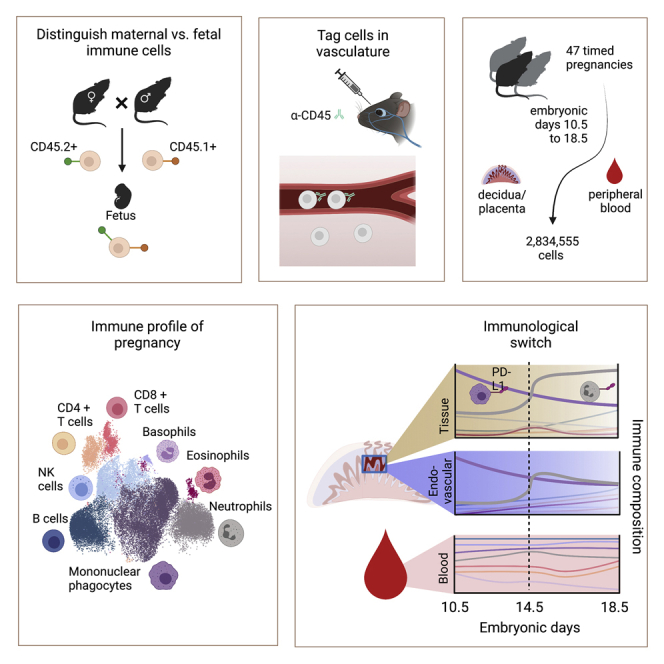

## Teaser

To better understand the immunology of pregnancy, Moore et al. establish a single-cell mass cytometry platform to deeply phenotype immune cells within the maternal-fetal interface. Their analysis highlights a potentially immunoregulatory role for the endovascular compartment of the maternal-fetal interface and localized maternal innate immune specialization throughout gestation.

## Introduction

Though transient, the placenta is a critical multifunctional organ. It mediates nutrients, gas, and waste exchange while simultaneously regulating maternal immune behavior to support tissue remodeling and to maintain tolerance (Ander et al., 2019). The placenta is the fetal contribution to the maternal-fetal interface (MFI), where the fetal chorion is anchored to modified maternal endometrium called the decidua, and where fetal trophoblasts are in direct contact with maternal blood (Ander et al., 2019; Hemberger et al., 2020). Immune regulation at the MFI is critical for pregnancy and healthy fetal development (Ander et al., 2019; Erlebacher, 2013; PrabhuDas et al., 2015). Despite this understanding, detailed knowledge of immune composition and regulation across pregnancy is sparse. A better understanding of immune dynamics and homeostasis is necessary to uncover pathogenic mechanisms and identify therapeutic interventions.

Immune cell phenotypes and function depend on their cellular interactions and microenvironment compartmentalization (Azizi et al., 2018; Schumacher et al., 2018). The MFI and tumor microenvironment share several similarities. Like an invasive tumor, placental architecture is complex and dynamic, requiring cell proliferation, tissue invasion, angiogenesis, vascular remodeling, and modulating tolerance (Holtan et al., 2009; Lala et al., 2021). As with tumor biology, the immune system plays an important role in these processes, facilitating and adapting to the ever-changing needs of gestation.

Considerable progress has been made in profiling the immune composition of the placenta. Recent meta-analysis of “bulk” transcriptomics (Yong and Chan, 2020) highlights low-resolution signatures of pregnancy complications. Flow cytometry, imaging studies (Tagliani et al., 2011; Arenas-Hernandez et al., 2015; Habbeddine et al., 2014; Kruse et al., 1999, 2002; Li et al., 2018; Rowe et al., 2012), and single-cell transcriptomics (Vento-Tormo et al., 2018) shed light on the relative abundance and phenotypes of selected cell types during pregnancy. Although the prior studies each contribute to our emerging understanding of pregnancy, the existing data lack the depth of analysis necessary to detect rare MFI cell types or resolve complex populations and their activation states at the single-cell level over time.

We developed an immune monitoring platform to interrogate the MFI throughout gestation using single-cell mass cytometry. We quantified 40 surface and intracellular markers across 2,834,555 cells of maternal and fetal origin from 47 mice over the course of 9 gestational days (embryonic days [E]10.5–18.5). By crossing congenic mouse strains and applying an injectable antibody to mark circulating cells, we were able to differentiate between maternal and fetal immune cells, as well as endovascular and tissue-resident cells. We detected an immunological cross-over point that coincides with a molecular switch point and immune reorganization driven by mononuclear phagocytes and neutrophils, many of which bear regulatory proteins like PD-L1.

We organized this dataset into a resource for dynamic immune cell composition at the MFI that can be easily mined for future experimental considerations. We further provide a framework to study and interrogate systemic immune perturbation in pregnancy that should have broad adaptability in future studies. Overall, our study reveals a surprisingly dynamic phenotypic diversity that low-dimensional methods and traditionally biased analyses have left concealed and should enable future cross-gestational and intercompartmental interrogations of the immune systems’ role in pregnancy.

## Results

### A distinct immune composition exists between placental endovascular and peripheral blood

**Figure 1:**
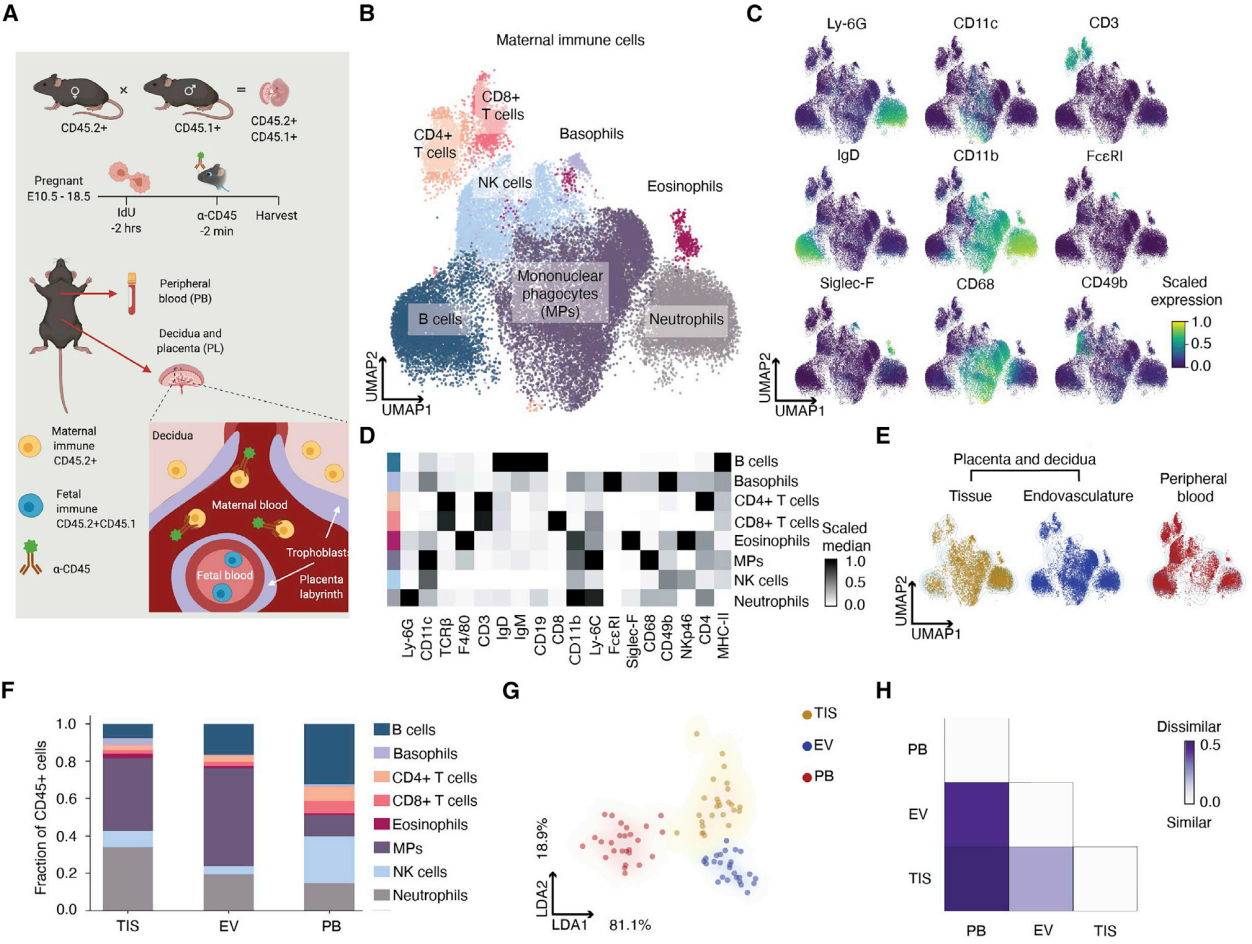
Single-cell mass cytometry reveals distinct immune composition between placental endovasculature and peripheral blood (A) Our setup distinguishes maternal from fetal immune cells and their localization in the vasculature of the MFI. (B and C) Composite UMAP of maternal immune cells in the MFI and PB across E10.5–E18.5, n = 26 mice. (C) Scaled cellular median intensity of lineage markers. (D) Scaled median expression of protein markers used for Leiden clustering across maternal immune cells. First column represents cell type. (E) Distribution of maternal immune cells across TIS, EV, and PB projected onto composite UMAP as contour plot. (F) Fraction of immune cells relative to total in each compartment. Aggregated embryonic days. (G) LDA based on maternal immune cell fractions in each compartment. Each dot represents a sample. (H) Bray-Curtis dissimilarity based on maternal immune cell fractions in each compartment.

Deep characterization of the immune content at the MFI is needed to elucidate tissue homeostasis and immune tolerance during pregnancy. Mice and humans have a hemochorial placenta and share similar decidual immune composition (Hemberger et al., 2020; Woods et al., 2018). We employed single-cell mass cytometry to map maternal immune cells at the MFI during the second half of mouse gestation using 40 markers (Table S1). We collected placentas from E10.5–E18.5 with intact decidua from C57BL/6 mice. Our ability to differentiate between maternal and fetal immune cells relied on a mating strategy crossing CD45.2 females and CD45.1 males, producing CD45.2+CD45.1+ fetal immune cells (Figure S1A). To partition maternal immune cells in the MFI by their tissue (TIS) and endovascular (EV) localization, pregnant mice were retro-orbitally injected with an anti-CD45 antibody (Figures 1A and S1B–S1D), labeling maternal immune cells in EV but not in TIS (Anderson et al., 2014; Tagliani et al., 2011). Maternal immune cells were found in the EV and TIS of independently processed decidua and placenta (Figure S1E). Cytometry results were confirmed by immunohistochemistry, showing maternal immune cells outside of CD31+ blood vessels in the placenta (Figure S1F). For the remainder of the analysis, we collected placentas with intact maternal decidua. Mass cytometry data were dimensionally reduced by UMAP and clustered with Leiden. Pooled organ and day data defined neutrophil, eosinophil, basophil, NK cell, B cell, T cell, and mononuclear phagocyte (i.e., monocytes, dendritic cells, and macrophages) clusters (Figures 1B and S1G). Cells were metaclustered based on their expression of common immune lineage markers (Figures 1C, 1D, and S1H). Basophils and eosinophils were split into separate clusters based on their FcεRI and c-Kit expression (Figures S1I–S1J). All cell populations were confirmed by traditional gating (Figure S1K).

Tissue microenvironments are known to regulate immune cell access and function. To broadly examine the impact of MFI microenvironments, UMAPs of composite data were overlaid with immune cells from maternal peripheral blood (PB), EV, or TIS (Figure 1E). Since maternal PB perfuses the MFI, we hypothesized that cells in EV would be more similar to PB than TIS. In contrast, cell profiles within EV appeared dramatically different from those in PB and TIS. We quantified compartment-specific proportions of each immune cell type (Figure 1F) to gain insight into the main cell types driving microenvironmental differences. Mononuclear phagocytes (MP) were the predominant cell type in the MFI, neutrophils were enriched in TIS, and T and B cells were enriched in PB. These intercompartmental differences were further evaluated by linear discriminant analysis (LDA; Figures 1G and S1L), where cell types were split by organ and compartment. EV was quantitatively more like TIS and strikingly dissimilar to PB. We also quantified the beta diversity (Bray-Curtis dissimilarity) between environments, replacing species with cell type identifiers (Horowitz et al., 2013). Our analysis revealed a higher similarity between EV and TIS than between EV and PB (Figure 1H), revealing a unique cellular niche in the EV space.

### Temporal emergence of fetal immune cells at the maternal-fetal interface

Human and mouse placentation precedes fetal immune cell development, reasonably guiding fetal immune characterization to be limited to the fetal body. Yet, evidence of fetal immune cells in umbilical cord blood (Jeanty et al., 2014) suggests their ability to travel to the MFI, and fetal cells have been found in other maternal tissues due to microchimerism (Kinder et al., 2017). As gestation progresses, it is reasonable to expect increasingly more fetal immune cells at the MFI.

**Figure 2:**
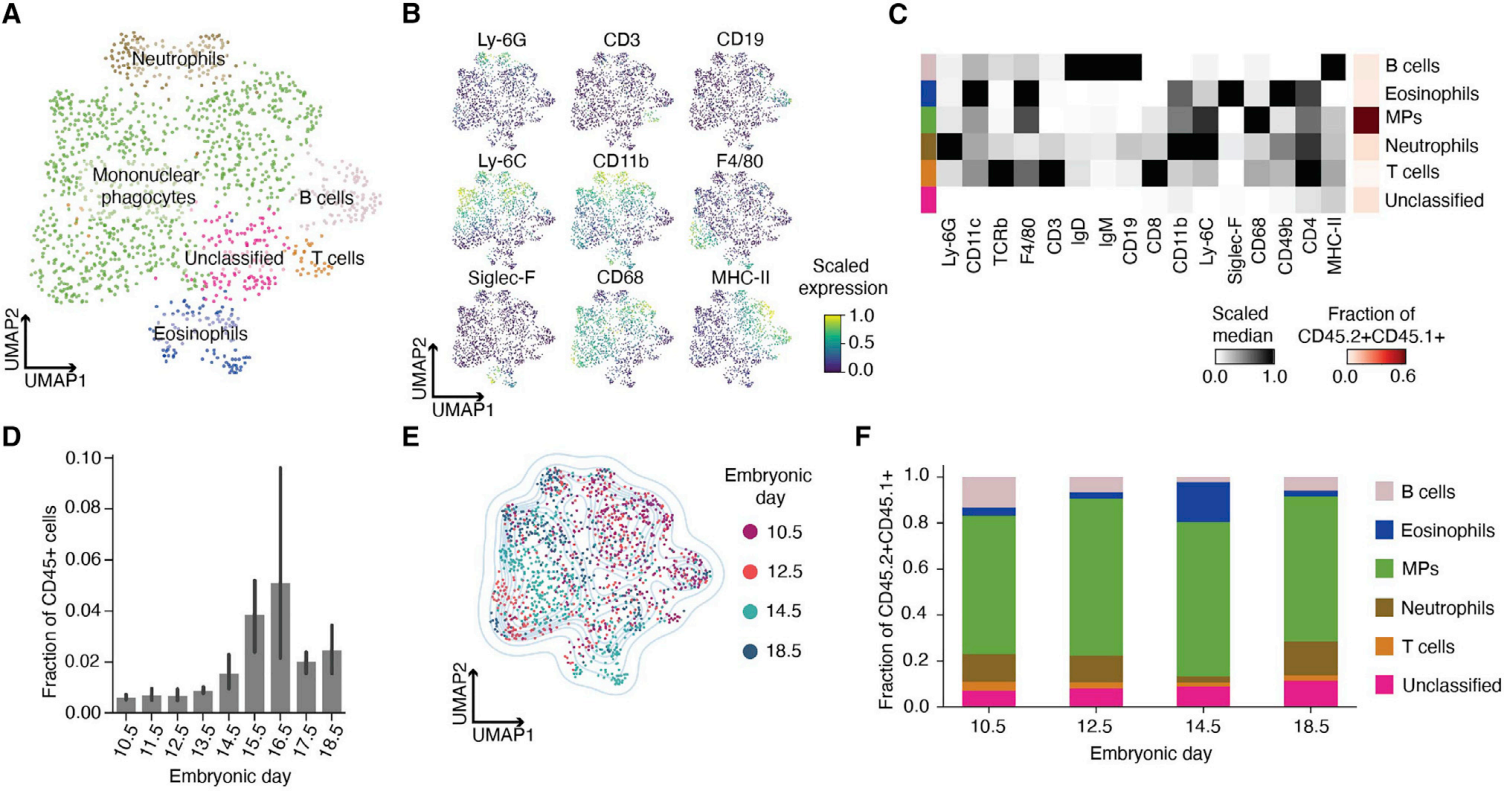
Fetal immune cell characterization at the maternal-fetal interface (A) Composite UMAP of fetal immune cells at MFI across E10.5 (n = 3), E12.5 (n = 3), E14.5 (n = 3), and E18.5 (n = 3). (B) Scaled cellular median intensity of lineage markers. (C) Scaled median expression of markers used for Leiden clustering. First column represents cell type. Last column represents cell fraction relative to total fetal immune cells. (D) Fraction of fetal immune cells relative to all immune cells at MFI across gestation. Samples by embryonic day, 10.5 (n = 3), 11.5 (n = 4), 12.5 (n = 10), 13.5 (n = 3), 14.5 (n = 7), 15.5 (n = 3), 16.5 (n = 3), 17.5 (n = 3), and 18.5 (n = 5). (E) Composite UMAP graph of fetal immune cells colored by embryonic day. (F) Fraction of fetal immune cells at E10.5, E12.5, E14.5, and E18.5.

Fetal immune cells (CD45.2+CD45.1+) were dimensionally reduced via UMAP and clustered with Leiden. Pooled fetal immune cells from E10.5, E12.5, E14.5, and E18.5 defined distinct clusters for MPs, neutrophils, eosinophils, B cells, and T cells (Figures 2A and S2A). We metaclustered these cells based on their expression of lineage markers (Figures 2B and S2B) and confirmed clustering using manual gating (Figure S2C). MPs represented more than 50% of the fetal immune cells in the pooled data (Figure 2C, red heatmap) but only made up ∼0.1%–0.8% of all immune cells (Figure 2D).

We noted a substantial increase in fetal immune cells over time (Figure 2D). To define the temporal changes in fetal immune composition, we colored the UMAP by gestational day (Figure 2E). The upper right quadrant of the UMAP was enriched with cells from E10.5 and E12.5. While cell types remained stable across gestation (Figure 2F), distinct subpopulations among neutrophil and MPs emerged over time (Figure S2D). By exhibiting temporal transitions in phenotype, these myeloid cells potentially fulfill distinct functions throughout gestation. Given that fetal monocytes are programmed to be functionally distinct from adult monocytes and have the potential to mount a protective, antimicrobial response (Krow-Lucal et al., 2014), the relative abundance of fetal MPs suggests a significant role at the MFI, especially later in gestation.

### Innate immune cell flux parallels maternal-fetal interface molecular dynamics

Tolerogenic mechanisms have been proposed regarding the innate immune system’s contributions at the MFI (Ander et al., 2019; Arck and Hecher, 2013; Chabtini et al., 2013; Mizugishi et al., 2015; PrabhuDas et al., 2015; Schumacher et al., 2018; Trowsdale and Betz, 2006). Mice without adaptive immune systems have normal numbers of healthy offspring (Burke et al., 2011; Guleria et al., 2005), but the depletion of innate immune cells can compromise fertility or pregnancy (Schumacher et al., 2018).

**Figure 3:**
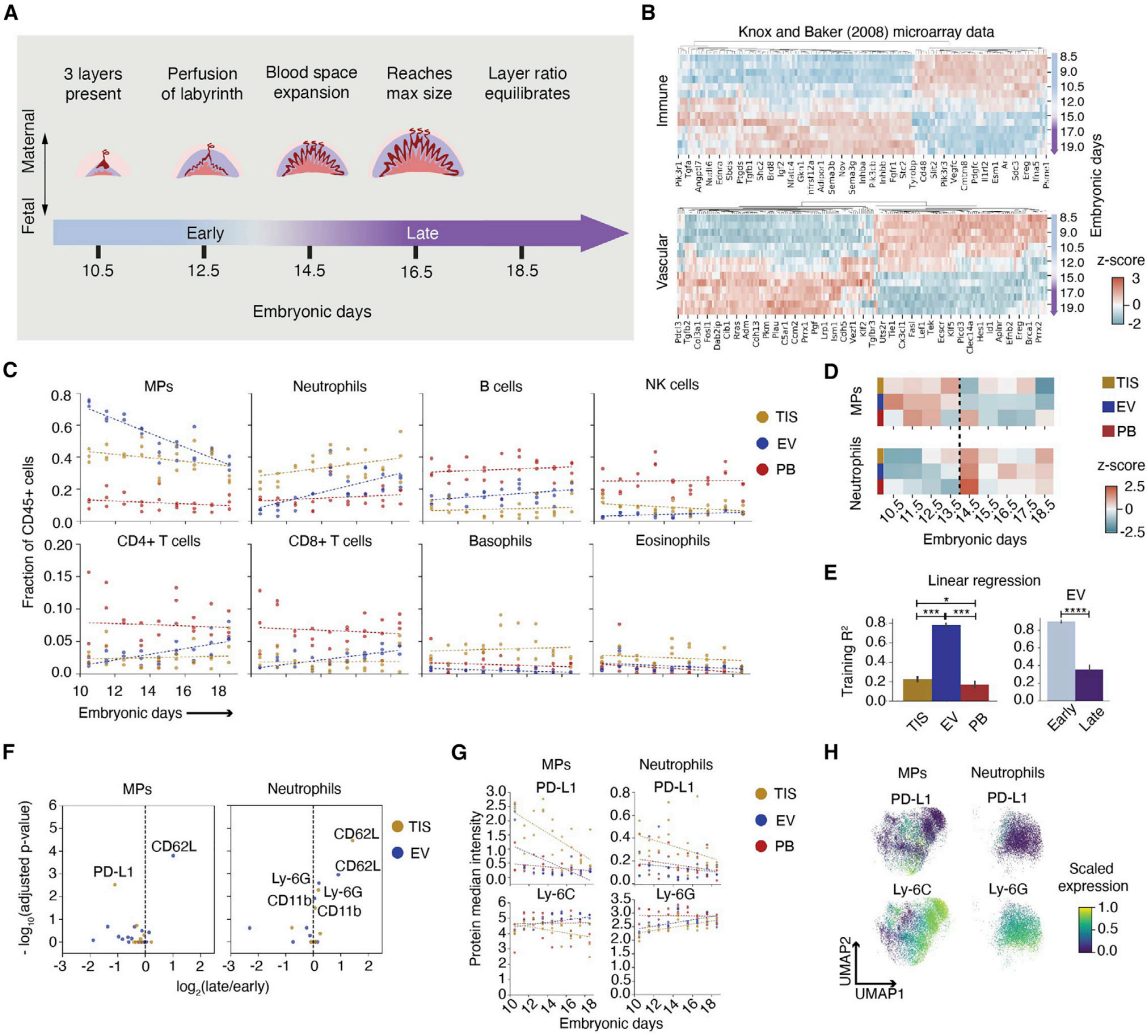
Mononuclear phagocytes and neutrophils define the gestational immune dynamics at the maternal-fetal interface (A) Stages of placental development throughout the last half of mouse gestation. (B) Microarray data from Knox and Baker (2008) analyzed for expression that significantly changed between E8.5 and 15.0. (C) Maternal immune cell fractions comparing TIS, EV, and PB from E10.5 to E18.5 fitted with linear generalized estimating equation (GEE). (D) Cell fraction of maternal MPs and neutrophils across embryonic days tested, colored by *Z* score. All days shown have n = 3, except for E12.5, which has n = 2. (E) Training R^2^ of linear regression across each compartment based on cell fractions across embryonic days (n = 26 per compartment). EV cell fractions were split into early (E10.5–E13.5, n = 11) and late (E14.5–E18.5, n = 15), and linear regression was run independently for each stage. ^∗^p ≤ 0.05, ^∗∗∗^p ≤ 0.001, ^∗∗∗∗^p ≤ 0.0001 (one-way ANOVA for comparing compartments, unpaired t test for early and late stages). (F) Volcano plots of protein median intensity in MPs and neutrophils between early and late stages across TIS and EV. Proteins with significant adjusted p values are shown. (G) Transformed median intensity of PD-L1 and Ly-6C in MPs, and PD-L1 and Ly-6G in neutrophils fitted with linear GEE across compartments and embryonic days. (H) Composite UMAPs of MPs and neutrophils colored by scaled expression of markers.

The mature mouse placenta is established at E10.5 and continues to grow in size and complexity until E18.5 (Figure 3A). Notably, E14.5 marks a rapid expansion of maternal blood space and maternal-fetal exchange to support rapid fetal growth until parturition (Coan et al., 2004, Coan et al., 2005). We assessed this transformation using publicly available gene expression data of mouse placenta (Knox and Baker, 2008), revealing 436 immune-related and 190 vascular-related genes that significantly changed before and after E14.5 (Figure 3B, Tables S2 and S3). To determine if this transcriptional switch is reflected at the cellular level, we turned to our mass cytometry data. Gestational day and fraction of immune cells were correlated for MPs (p < 0.0001) and neutrophils (p < 0.0001) in EV (Figure 3C, Table S4). MPs exhibited a decreasing trend while neutrophils showed an increasing trend. An increase in immune cell abundance emerged in EV at E14.5 (Figure S3A), indicating immune enrichment in the decidua and/or placenta, although blood space expansion is restricted to the placenta. MPs are enriched in EV throughout gestation, but neutrophils were TIS biased only at early timepoints (Figure S3B). MPs and neutrophils remained the most abundant immune cell types at the MFI across gestation (Figure 3C). Given their requirement for successful pregnancy (Schumacher et al., 2018), these data suggest their important role in innate immune homeostasis at the MFI.

To highlight differences in immune cell contribution over time, we calculated the *Z* score of cell-type abundance across embryonic days within each compartment (Figure 3D). Neutrophils and MPs exhibit a cross-over point between E13.5 and E14.5 in EV, suggesting a coordinated and reciprocal change in relative abundance. We used linear regression to determine if embryonic day could be predicted by immune cell abundance (Figures S3C and S3D). EV showed reliable gestational dynamics that predicted gestational age (Figure S3E). Neutrophil and MP abundance alone were also able to accurately predict gestational day (Figures 3E, S3F, and S3G). To determine whether this predictability was consistent across gestation, we analyzed early (E10.5–E13.5) and late (E14.5–E18.5) gestation independently for all immune cells (Figures S3H and S3I) or only neutrophils and MPs (Figures S3J and S3K). Using all immune cells, we found that both periods mapped equally well (Figure S3G, EV). When only using neutrophil and MP abundance, early gestational timepoints mapped 2.5-fold (p < 0.0001) more reliably than late gestational timepoints (Figure 3E, EV). This suggests that the relationship between these two cell types is critical in early stages of pregnancy.

In summary, we show pronounced molecular and cellular transitions that occur during a period of rapid blood space expansion at the MFI. Our data suggest that changes in EV cell profiles might be a significant contributor to the immune transcriptomic changes in the MFI during the observed switch (Knox and Baker, 2008; Figure 3B).

### Mononuclear phagocytes and neutrophils distinguish unique states in early and late gestation

As the MFI develops to accommodate growing fetal demands, its changing microenvironments may influence MP and neutrophil functional states. Differential protein expression in early and late gestation was examined in MPs and neutrophils across compartments (Figure 3F). Increased CD62L expression on EV MPs and neutrophils, and in TIS neutrophils, suggests increased recruitment in late gestation. Interestingly, immune checkpoint protein PD-L1 on TIS MPs significantly decreased over time, suggesting less need for immune suppression in late gestation, as parturition nears.

Given the increasing interest in myeloid expression of checkpoint proteins (Strauss et al., 2020), we evaluated lineage markers and PD-L1 expression across gestation on MPs and neutrophils (Figure 3G, Table S5). Ly-6C, a lineage and pro-inflammatory marker in MPs (Guilliams et al., 2018), decreased throughout gestation in TIS MPs, almost reaching significance (p = 0.055). PD-L1 expression significantly decreased over time in EV (p = 0.022) and TIS (p < 0.0001), suggesting a response to changing needs between early and late gestation. The average median expression of Ly-6C was 1.11-fold lower in TIS compared with PB (p = 0.004; Figure 3G). In contrast, PD-L1 expression was 0.25-fold higher in TIS compared with PB (p < 0.0001), suggesting phenotypic adaptation to the MFI microenvironment.

We repeated these analyses for neutrophils (Figure 3G) and found a significant increase in Ly-6G expression over time for EV (p = 0.002) and TIS (0.003), suggesting more mature neutrophils (Xie et al., 2020) in the MFI in late gestation. There was a downward trend in PD-L1 expression on neutrophils across all compartments. The average median expression of Ly-6G was 1.09-fold lower in EV (p = 0.0001) and 1.16-fold lower in TIS (p < 0.0001) compared with PB. PD-L1 expression was 0.52-fold higher in TIS compared with PB (p < 0.0001). These data suggest an inverse relationship between neutrophil maturity and immunosuppressive potential, which aligns with observations made in the tumor microenvironment (Mackey et al., 2019).

Given the significant effects of gestational day on protein expression, either global changes or a temporal flux of phenotypic subsets could be contributing factors. We examined the expression patterns of Ly-6C and PD-L1 on MPs, and Ly-6G and PD-L1 on neutrophils across single cells in a UMAP (Figure 3H). MPs showed a range in Ly-6C and multiple PD-L1-positive regions. The neutrophil UMAP showed a gradient of increasing Ly-6G expression and a single PD-L1-positive region. This differential protein expression expands the possibility of a diverse myeloid compartment that remodels as a function of microenvironment and gestational day.

### Diverse and dynamic PD-L1+ mononuclear phagocytes are unique to the maternal-fetal interface

**Figure 4:**
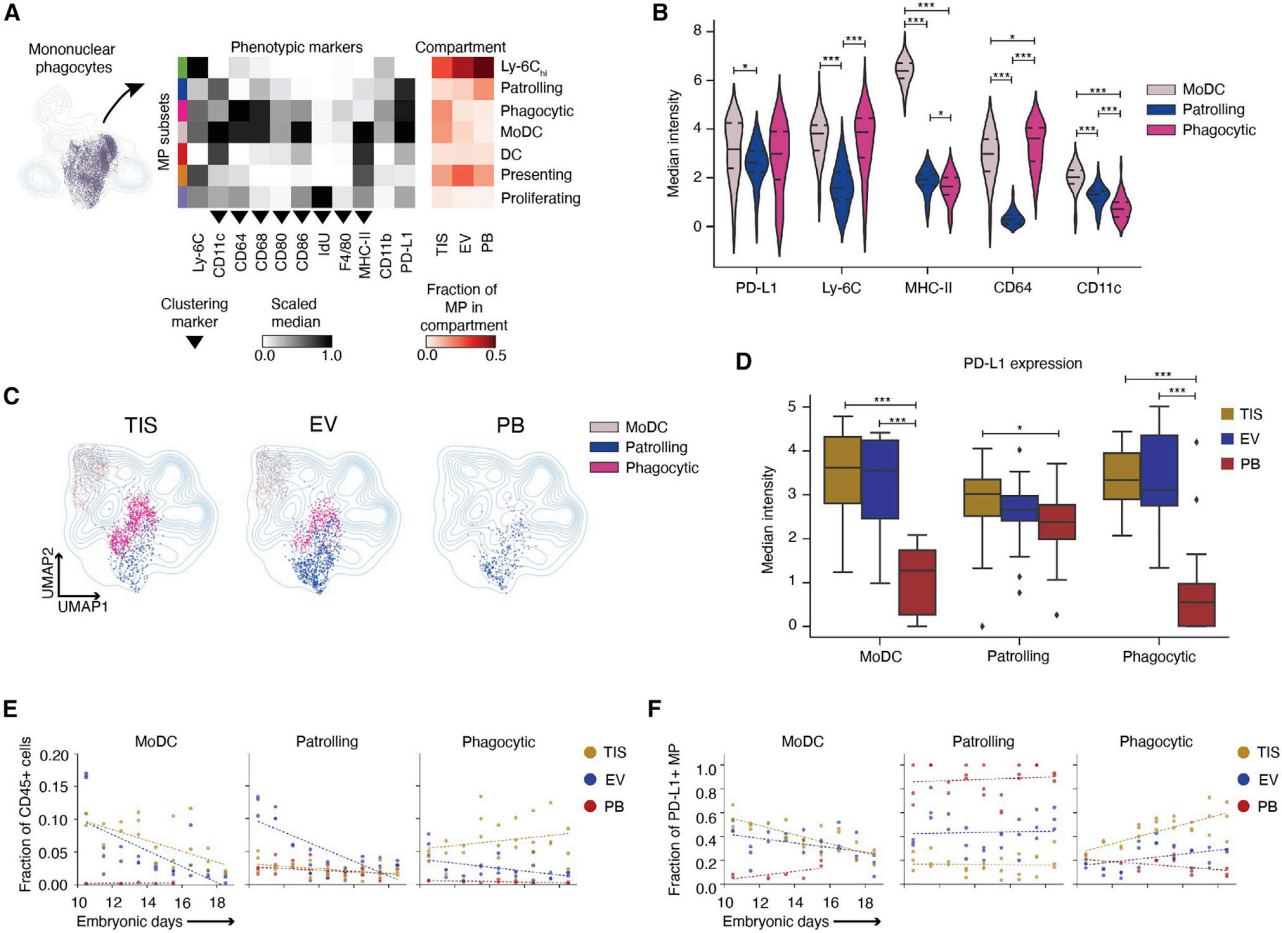
Phenotype specialization and temporal regulation of mononuclear phagocytes to placenta microenvironment (A) UMAP and Leiden re-clustering of MPs. Scaled medians of marker expression show seven subsets. The last three columns show the fraction of each MP subset relative to all MPs across compartments (n = 26 per compartment). (B) Transformed median intensities across PD-L1+ MP subsets. (C) UMAPs showing distribution of PD-L1+ MP subsets across compartments. (D) Transformed PD-L1 median intensity of moDC, patrolling, and phagocytic subsets across compartments. (E) Linear GEE fitted fractions of moDC, patrolling, and phagocytic MP subsets relative to maternal immune cells across compartments and gestation. (F) Linear GEE fitted fractions of PD-L1+ MP subsets out of all PDL1+ MPs across compartments and gestation. For (B) and (D), significance is shown as ^∗^p ≤ 0.05, ^∗∗∗^p ≤ 0.001 (one-way ANOVA per marker in B and per cell type in D).

Monocytes are traditionally dismissed as undifferentiated precursors to dendritic cells and macrophages, resulting in their under-explored function in pregnancy. To assess MP functional heterogeneity at the MFI, we re-clustered MPs using IdU (proliferation), CD11c, F4/80, CD86, CD80, CD64, CD68, and MHC-II (Figures 4Aand S4A). We identified 11 subsets of MPs and assigned them identities based on marker expression (Figures S4B and S4C). Eight out of 11 subsets were present across compartments (Figures S4D–S4E). We metaclustered subsets by phenotypic similarity, resulting in eight subsets, seven of which were present in all compartments (Figure 4A). We confirmed subsets using canonical gating strategies and increased their resolution using CD11c expression (Figure S4F).

A high expressing Ly-6C subset occupied up to ∼50% of all MPs, suggesting a majority monocyte population (Figure 4A, red heatmap). We further divided MPs by Ly-6C (hi)gh, (int)ermediate, and low, which are traditionally used to categorize monocytes as classical, intermediate, and non-classical monocytes, respectively (Figure S4G). Subsets traditionally assigned as classical or non-classical included notable cell fractions of all three Ly-6C levels (Figure S4H), suggesting conventional classifications can obscure their biology at the MFI. It is important to note that Ly-6C low-cells in our MP analysis also include macrophages and dendritic cells.

Immune checkpoint ligand, PD-L1, was highly expressed in three MP subsets (Figure 4A), each distinct based on the expression of functional markers (Figure 4B). The CD64 high PD-L1+ subset was named “phagocytic,” as CD64 is involved in phagocytosis, and the highest MHC-II-expressing subset was named “moDC,” as it is most likely a monocyte-derived dendritic cell. The remaining PD-L1 subset was named “patrolling” as it was low in Ly-6C, MHC-II, CD64, and adhesion protein CD11c, indicating surveillance. Based on their cell frequency, phagocytic and moDC were TIS enriched (Figure 4A, red heatmap). UMAP contour maps were overlaid with each compartment’s PD-L1 subsets to interrogate distribution. Patrolling MPs were present across compartments, while moDC and phagocytic MPs, like most subsets, were largely restricted to the MFI (Figures 4C and S4D).

Monocytes adapt to their microenvironment, carrying out distinct functions based on EV or TIS localization (Auffray et al., 2007; Carlin et al., 2013; Nahrendorf et al., 2007). Since patrolling MPs were present in all compartments, we evaluated their phenotypic changes as they moved from PB to the MFI. Patrolling MPs in TIS had significantly higher PD-L1 median expression than those in PB (1.3-fold, p = 0.037; Figure 4D), consistent with patterns observed in Figure 3G. This trend was even more pronounced in moDC (3.2-fold, TIS to PB, p = 0.001) and phagocytic (3.7-fold, TIS to PB, p = 0.001), although cells in these subsets are less abundant in PB. PD-L1 expression has been reported to be restricted to non-classical and intermediate monocytes (Bianchini et al., 2019). However, we found that while classical monocytes in PB lacked PD-L1, PD-L1 expression was significantly upregulated at the single-cell level upon TIS entry (Figure S4I). There was also a higher fraction of classical monocytes that expressed PD-L1 in TIS compared with PB (Figure S4J).

We next asked if MP subsets emerged at different points of gestation (Figures 4E and S4K). On average, moDC and phagocytic MPs had a significant TIS bias compared with PB (moDC: p < 0.0001, phagocytic: p < 0.0001). However, over time moDCs significantly decreased in EV (p = 0.0001) and TIS (p < 0.0001), while phagocytic MPs increased in TIS. Patrolling MPs were only enriched “early” in EV, decreasing significantly over time (p < 0.0001; Figure 4E, Table S6). MoDC and patrolling MPs exhibited a short-lived EV bias at E10.5, confirming previous reports (Kruse et al., 1999).

As a fraction of PD-L1-expressing MPs, moDCs were consistently enriched in the MFI compared with PB (TIS: p < 0.0001, EV: p < 0.0001; Figure 4F, Table S7). Phagocytic MPs also showed an MFI bias throughout gestation compared with PB (TIS: p < 0.0001, EV: p = 0.002). Over time, moDCs significantly decreased in TIS (p < 0.0001) and EV (p = 0.0008), while phagocytic MPs increased (EV: p < 0.0001, TIS: p < 0.0001). Patrolling MPs remained consistent and accounted for the majority of PD-L1+ MPs in PB (Figure 4F), although were a much smaller fraction than the Ly-6C_hi_ PB dominant MP subset (Figure S4L, Table S8). These data suggest that moDC and patrolling MPs might serve important regulatory roles establishing the mature MFI, while phagocytic MPs potentially assist in tissue remodeling and other homeostatic processes throughout gestation.

Collectively, these data show increased heterogeneity of MP cell states and PD-L1 phenotypic expansions within the MFI, likely due to its unique microenvironments. These specialized subsets exhibit unique temporal dynamics that align with structural changes at the MFI that necessitate regulatory MPs that can function in tissue remodeling.

### Noncanonical neutrophils in early gestation placenta bear PD-L1 and proliferate *in situ*

**Figure 5:**
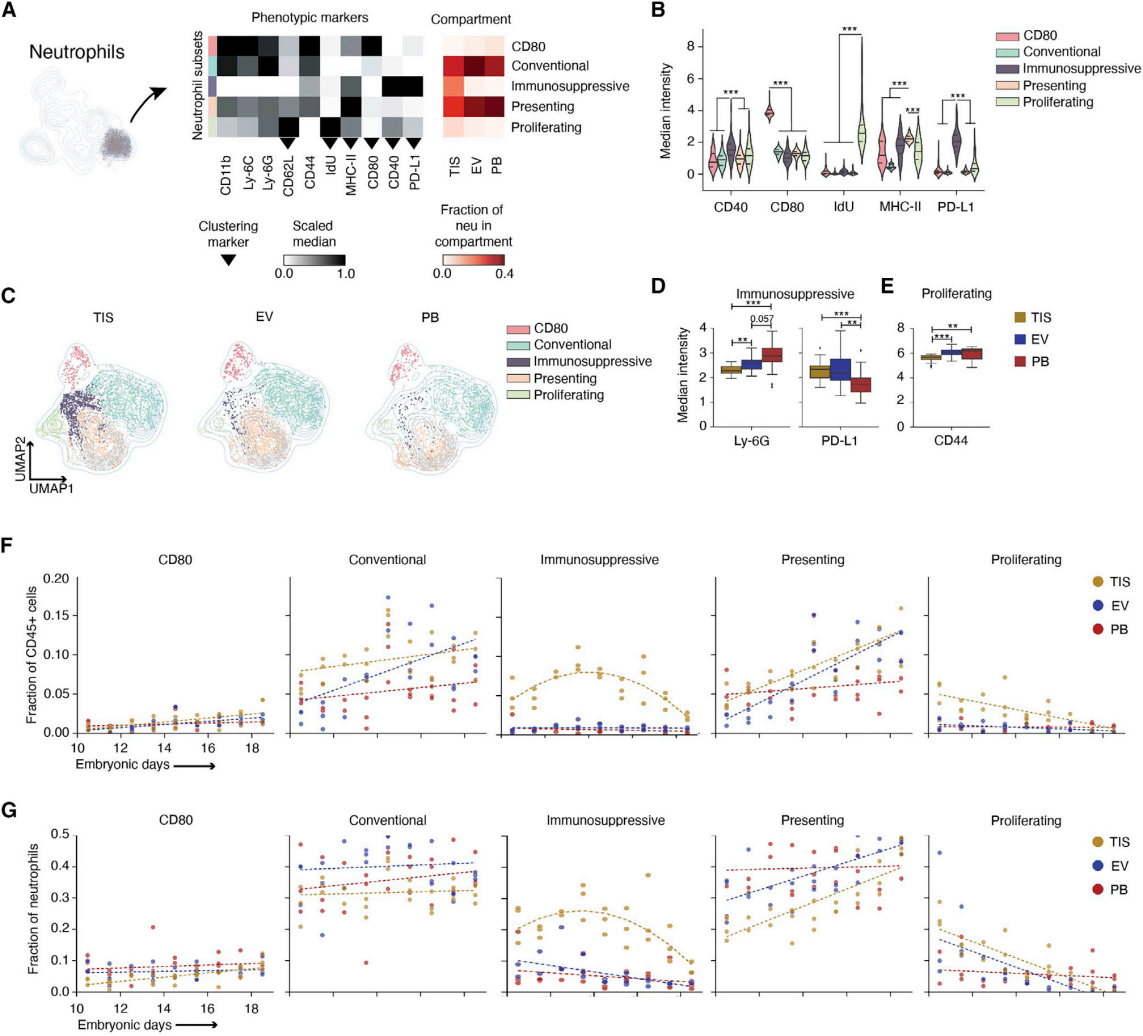
Placenta enriches for noncanonical neutrophil subsets in tissue-compartment-specific manner (A) UMAP and Leiden re-clustering of neutrophils. Scaled medians of marker expression show five neutrophil subsets. The last three show the fraction of each subset relative to all neutrophils across compartments (n = 26 per compartment). (B) Transformed median intensities across subsets. (C) UMAPs showing distribution subsets across compartments. (D) Transformed Ly-6G and PD-L1 median intensity of the immunosuppressive subset across compartments. (E) Transformed CD44 median intensity of the proliferating subset across compartments. (F) Linear GEE fitted to CD80, conventional, presenting, and proliferating subsets relative to maternal immune cells across compartments and gestation. GEE was applied to fit a quadratic model to the immunosuppressive subset. (G) Linear GEE fitted to CD80, conventional, presenting, and proliferating subsets relative to total neutrophils across compartments and gestation. GEE was applied to fit a quadratic model to the immunosuppressive subset. For (B), (D), and (E), significance is shown as ^∗∗^p ≤ 0.01, ^∗∗∗^p ≤ 0.001 (one-way ANOVA per marker in B and per cell type in D and E).

While remarkably diverse, neutrophils are often characterized as an injurious monolith, commonly associated with pregnancy complications (Giaglis et al., 2016; Tong and Abrahams, 2020). Our data show neutrophils as an abundant cell type at the healthy MFI throughout gestation (Figure 3C), suggesting an important homeostatic role in pregnancy. Our protein analysis revealed a PD-L1+ neutrophil subset (Figures 3F–3H), suggesting their regulatory potential. Our neutrophil dataset revealed expression of regulatory immune markers typically associated with MP function. We re-clustered neutrophils based on CD80, CD62L, CD40, MHC-II, PD-L1, and IdU (Figures 5A and S5A–S5C). Eleven subsets that spanned compartments were generated by Leiden (Figures S5D and S5E) and metaclustered into five subsets based on phenotypic similarity. We classified the subsets as “presenting,” “immunosuppressive,” “proliferating,” “CD80,” and “conventional” due to their respective MHC-II (Lin and Loré, 2017; Vono et al., 2017), PD-L1 (Wang et al., 2017), IdU, CD80, and canonical expression profiles (Figure 5B). These subsets were confirmed using canonical gating strategies (Figure S5F). We found neutrophils expressing relatively high MHC-II across immunosuppressive, proliferating, and CD80 subsets, and a subset of co-stimulation protein CD40-expressing cells in conventional and presenting (Figures S5B and S5C). PB was enriched with the CD40-negative fraction of the conventional and presenting subsets, while the MFI had a uniform distribution (Figures 5C, S5B, and S5D).

Data merged by day showed conventional and presenting as most abundant, and immunosuppressive and proliferating as TIS enriched (Figure 5A, red heatmap, and 5C). The remaining subsets were enriched in EV and PB. Since immunosuppressive and proliferating had a TIS bias, we asked how both subsets differed phenotypically in EV and PB. As the immunosuppressive neutrophils moved from PB to EV, their PD-L1 expression increased 1.4-fold (p = 0.006); Ly-6G expression, used to assess neutrophil maturity (Xie et al., 2020), decreased 1.12-fold (p = 0.06; Figure 5D). There was a further 1.1-fold (p = 0.01) decrease in Ly-6G as PD-L1-expressing neutrophils moved from EV to TIS. Immune cells in the tumor microenvironment can regress in maturity as a form of immune regulation (Mackey et al., 2019).

Mature neutrophils are mitotically inactive with cell-cycle arrest, but in cancer, neutrophil granulopoiesis can occur outside of the medullary spaces of the bone marrow (Mackey et al., 2019). With the numerous parallels between tolerance mechanisms in tumor immunology and pregnancy, it is reasonable that neutrophil precursors could be seeding the MFI and participating in local granulopoiesis. CD40+ proliferating neutrophils decreased in homing marker CD44 expression (1.07-fold, p = 0.005) as they moved from PB to TIS (Figure 5E) and were significantly enriched in the MFI (Figures 5A, red heatmap, and 5C).

We next interrogated the population dynamics across gestation. CD80 was consistently the least abundant neutrophil subset throughout gestation. Conventional and presenting remained the most abundant subsets across compartments and showed MFI bias at late timepoints (Figure 5F, Table S9). On average, conventional was higher in the MFI compared with PB (EV: p = 0.01, TIS: p < 0.0001), as was presenting (TIS: p = 0.001). Immunosuppressive (p < 0.0001) and proliferating (p < 0.0001) demonstrated TIS bias compared with PB. CD80 significantly increased across compartments over gestation (TIS: p = 0.006, EV: p = 0.0008, PB: p = 0.02). Conventional significantly increased in blood-rich compartments (EV: p = 0.005, PB: p = 0.03), while presenting’s significant increase was limited to the MFI (TIS: p = 0.0009, EV: p < 0.0001). The proliferating subset significantly decreased (p < 0.0001) in TIS over gestation. Tissue-localized immunosuppressive neutrophils exhibited a negative parabolic-shaped trend during gestation and required a nonlinear fit. There was a significant quadratic effect between gestational day and immunosuppressive neutrophils in TIS (p < 0.0001), showing an initial rise between E10.5 and E13.5 and then a gradual decrease at later timepoints.

As a fraction of neutrophils, conventional and presenting were the dominant subsets in PB and EV for most of gestation (Figure 5G). Immunosuppressive (p < 0.0001) and proliferating (p = 0.001) maintained a TIS bias when compared with PB (Figure 5G, Table S10). There was a significant increase in presenting in EV (p = 0.009) and TIS (p = 0.001) and a decrease in proliferating in EV (p = 0.007) and TIS (p < 0.0001) over gestation. The nonlinear relationship between tissue-localized immunosuppressive neutrophils and gestational day reappeared, exhibiting a significant quadratic effect (p < 0.0001). Neutrophil subsets showed clear and distinct trends during gestation, demonstrating notable temporal dynamics that suggest neutrophil responsiveness to a changing environment during pregnancy.

### Response to poly(I:C) challenge is gestationally dependent, reducing placental PD-L1+ cells

**Figure 6:**
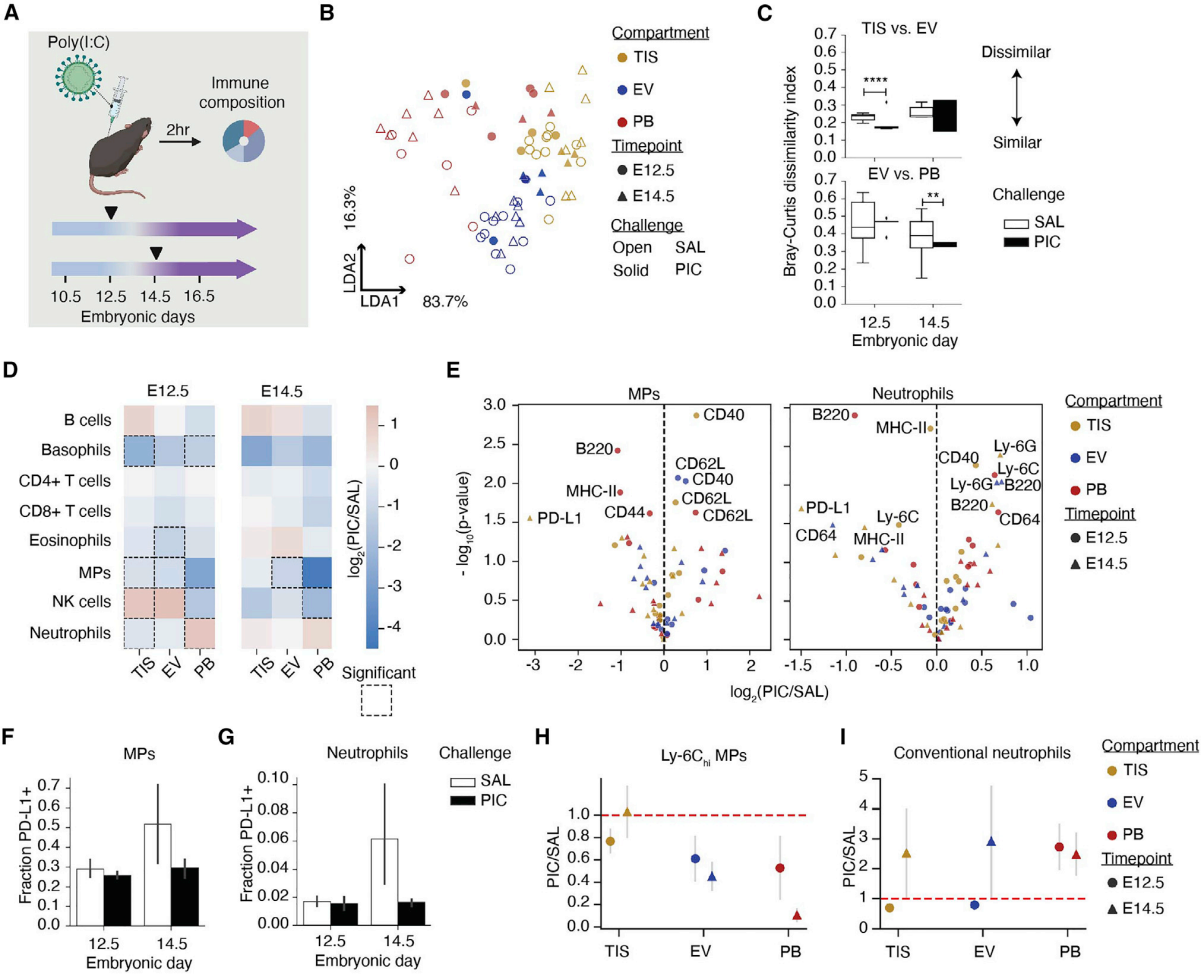
Immune response to systemic perturbation is dependent on gestational day (A) Set up of systemic maternal immune challenge with poly(I:C) (PIC): baseline E12.5 TIS (n = 10), EV (n = 10), PB (n = 6); baseline E14.5 TIS (n = 7), EV (n = 7), PB (n = 7); PIC E12.5 TIS (n = 4), EV (n = 4), PB (n = 4); PIC E14.5 TIS (n = 3), EV (n = 3), PB (n = 3). (B and C) LDA (B) and Bray-Curtis (C) dissimilarity based on maternal immune cell fractions in each compartment and by treatment. ^∗∗^p ≤ 0.01, ^∗∗∗∗^p ≤ 0.0001 (unpaired t test). (D) Maternal immune cell fraction was compared by taking the log_2_(PIC/SAL). Significant (p < 0.05) changes between challenges are encased by a dotted line. (E) Volcano plots of protein median intensity changes in MPs and neutrophils by compartment following PIC. Proteins with significant changes between SAL and PIC are shown. (F) PD-L1+ fraction of MPs at E12.5 and E14.5 with SAL or PIC. (G) PD-L1+ fraction of neutrophils at E12.5 and E14.5 with SAL or PIC challenge. (H) The PIC over SAL counts of Ly-6C_hi_ MP subset and conventional neutrophil subset were analyzed between days and compartments. p values are uncorrected for multiple comparisons in (D) and (E).

Systemic immune activation in early pregnancy can dysregulate homeostatic and tolerogenic mechanisms at the MFI, increasing the risk of pregnancy and post-natal complications (Yockey and Iwasaki, 2018). We applied our immune monitoring framework to examine the maternal response 2 hours after the systemic administration of poly(I:C), a viral antigen mimic. Specifically, we compared immune responses in the MFI and PB compartments across the transition from early (E12.5) to late (E14.5) gestation (Figure 6A). Intercompartmental differences following poly(I:C) were evaluated by LDA using cell abundance (Figure S6A). Each data point represents a baseline (open symbol) or poly(I:C)-treated (solid symbol) animal at E12.5 (circle) or E14.5 (triangle, Figure 6B). While poly(I:C)-treated TIS remained largely unchanged, the PB and EV profiles were substantially perturbed relative to saline and mapped more closely to the TIS cell profiles. This suggests a significant disruption in PB and EV populations following poly(I:C), but a relative stability in the TIS cell profile. The calculated beta diversity measures (Figures 6C and S6C) following poly(I:C) confirmed a significant increase in similarity between EV and TIS at E12.5 (1.13-fold change, p < 0.0001) and between EV and PB at E14.5 (1.2-fold change, p = 0.002).

To determine the immune cell populations that mediated the poly(I:C)-induced changes, we calculated cell frequency (Figure S6B) and summarized the results with log-fold change of major cell types across compartments (Figures 6D and S6D). At E12.5, poly(I:C) resulted in neutrophil enrichment in PB and MP depletion at the MFI, suggesting compartmentalized responses and the MFI’s ability to continue to regulate cell access from perfusing maternal blood. Poly(I:C) at E14.5 resulted in MP depletion in EV. Coinciding with the gestational cross-point (Figure 3) and vulnerable periods of pregnancy in humans (Allanson et al., 2010; Srinivas et al., 2006) and mice (Carpentier et al., 2011, 2013; Hsiao and Patterson, 2011; Vermillion et al., 2017), we see responses to poly(I:C) being dependent on gestational age with a higher magnitude of responses occurring earlier in gestation (Figure 6D). The poly(I:C)-dependent perturbation of MP (Figure S6E) and neutrophil (Figure S6F) subsets also depended on gestational day and compartment.

Since MPs and neutrophils are the innate immune sensors for pathogens, we examined how expression of functional proteins changed after poly(I:C). There were seven significantly changed activation molecules in MPs and neutrophils with MHC-II, B220, CD40, and PD-L1 in common (Figure 6E). Considering PD-L1-expressing MP and neutrophil subsets contracted following poly(I:C) (Figures S6E and S6F), and PD-L1 was the most significantly changed molecule in tissue-localized MPs and neutrophils (Figure 6E), we quantified the fraction of PD-L1-expressing cells in both populations (Figures 6F and 6G). Poly(I:C) resulted in a 1.8-fold reduction of PD-L1-expressing MPs at E14.5, which mostly impacted the proliferating subset (Figures 6F and S6G). Neutrophils had a 3.7-fold reduction at E14.5, which reduced the conventional and proliferating subsets (Figures 6G and S6H). Poly(I:C) also decreased the median expression of PD-L1 across every MP subset except phagocytic and presenting (Figure S6I), and every neutrophil subset except presenting in TIS at E14.5 (Figure S6J). However, poly(I:C) increased PD-L1 expression among MP subsets in PB and EV, perhaps indicating that poly(I:C) mobilized PD-L1-expressing neutrophils and MPs out of TIS.

Given the temporal and compartmental differences in PD-L1+ MPs and neutrophils, we interrogated how the most abundant and potentially inflammatory subpopulations responded to poly(I:C). Ly-6C_hi_ MPs contracted in PB and EV, notably at E14.5 (Figures 6H and S6E). Conventional neutrophils experienced an expansion in PB, but only on E14.5 in the MFI (Figures 6I and S6F). These findings show that even 2 hours after poly(I:C), the MFI can respond rapidly and uniquely depending on the compartment and gestational day.

We next examined whether fetal MPs responded similarly to maternal MPs following the systemic perturbation. Fetal MPs significantly contracted 1.4-fold (p = 0.004) at the MFI on E14.5, but not on E12.5 (Figure S6K), whereas maternal MPs showed a significant reduction on both days. These data suggest that the fetal immune response in the placenta is also dependent on gestational day.

Overall, tissue-localized PD-L1-expressing MPs and neutrophils were the most modulated subsets following poly(I:C). Even when the TIS compartment of the MFI remained largely unchanged (Figure 6D, E14.5), we see shifts away from PD-L1-bearing cells (Figures 6F and 6G, E14.5) that may contribute to loss of tolerance in immune activation and allogeneic scenarios.

## Discussion

We mapped gestational immune dynamics of the MFI to identify cell state, abundance, and localization using single-cell mass cytometry. Our strategy (Figure 1) captured maternal and fetal immune cells and discerned maternal immune cells in maternal PB and the MFI endovascular (EV) and tissue (TIS) compartments. PB and EV immune profiles substantially diverged from one another (Figures 1G and 1H). Given that expression of cell adhesion molecules lining decidual vasculature changes throughout gestation (Kruse et al., 2002), we speculate that the selective engagement of the EV endothelium drives this divergence.

The gestational dynamics observed for fetal (Figure 2) and maternal (Figure 3) innate immune cells within the MFI captured adaptations similar to tumor biology (Holtan et al., 2009; Lala et al., 2021). We highlighted PD-L1-expressing MPs and neutrophils and their transience in MFI as significant candidates in mediating immune tolerance at the MFI. Neutrophils and MPs appeared to have tight coordination, displaying an inverse relationship over time and a gestational cross-over point between E12.5 and 14.5 (Figures 3B–3D) that aligns with vascular remodeling and expansion (Figure 3A) and with the period of susceptibility to immune-related pregnancy complications (Allanson et al., 2010; Carpentier et al., 2011, 2013; Hsiao and Patterson, 2011; Srinivas et al., 2006).

Our results uncover an MFI adaptation of immunomodulatory MPs (Figure 4) and neutrophils (Figure 5) that may contribute to immune tolerance. We identified seven distinct MP subsets at the MFI, three of which were PD-L1+ (Figure 4). As deletion of PD-L1 in dendritic cells results in enhanced antitumor immunity (Oh et al., 2020), our data suggest that PD-L1+ subsets could be modulating adaptive immunity and supporting placental growth. Furthermore, PD-L1 monocyte-derived subsets have been reported to support other functions related to placentation, including the early response to tissue damage (i.e. remodeling) and scavenging debris (Bianchini et al., 2019; Olingy et al., 2017). Given the broad role of MPs in immune regulation, tissue homeostasis, and remodeling, it is reasonable that abundance and localization of unique PD-L1 subsets in the MFI are strongly linked to gestation.

We identified five neutrophil subsets in the MFI (Figure 5A), demonstrating heterogeneity associated with immune tissues (Evrard et al., 2018; Grieshaber-Bouyer and Nigrovic, 2019; Xie et al., 2020) and tumors (Sagiv et al., 2015). In pregnancy, the role of neutrophils has been limited to placental damage and preterm labor (Giaglis et al., 2016; Tong and Abrahams, 2020), yet their depletion exasperates injury (Higashisaka et al., 2018). The neutrophil heterogeneity captured in our study implies functional diversity, where its disruption could inhibit protective functions.

The proliferating neutrophils (Figures 5F and 5G) at the MFI might be related to the increased frequency of immature granulocytes in PB during pregnancy (Blazkova et al., 2017). Like tumors (Wu et al., 2018), the healthy placenta releases neutrophil/monocyte chemoattractant CCL2 (Carpentier et al., 2011) for recruitment to the MFI. Proliferating granulocytes are not typically found outside the bone marrow (Mackey et al., 2019), but neutrophil precursors can seed distant tissues to support tumor growth (Kowanetz et al., 2010; Wu et al., 2018). The CD40+ and PD-L1+ proliferating subset could be expanding in the MFI to regulate T cell, macrophage, and NK cell activity (Rakhmilevich et al., 2012).

The PD-L1+ neutrophils at the MFI may complement the role of immune-suppressive myeloid-derived suppressor cells (Ahmadi et al., 2019). Regulatory effects of PD-L1+ neutrophils have been shown in cancer and microbial infections (Bowers et al., 2014; Castell et al., 2019; Chun et al., 2015; de Kleijn et al., 2013; Langereis et al., 2017; Mcnab et al., 2011; Schulte-Schrepping et al., 2020). Tumor-induced PD-L1 upregulation on neutrophils increases neutrophil lifespan (Cheng et al., 2018), enabling them to exert suppressive potential longer (He et al., 2015; Wang et al., 2017). Given that proliferating and PD-L1+ neutrophil subsets had significant early enrichment in TIS (Figures 5F and 5G), they might be supporting placenta immune evasion and development.

PD-L1 expression in the MFI has reportedly been restricted to trophoblasts, potentially allowing the fetus to inactivate maternal immune cells (Gong et al., 2014; Guleria et al., 2005; Petroff et al., 2003; Veras et al., 2017). PD-L1-expressing immune cells were recently reported in the MFI, but the analysis was limited to decidual T and NK cells (Meggyes et al., 2019). Given that PD-L1 checkpoint blockade has been associated with pregnancy loss (Zeng et al., 2020) and neutrophil depletion with pregnancy complications (Nadkarni et al., 2016), our data imply a new role for PD-L1+ myeloid cells at the MFI.

Systemic immune activation during pregnancy has been shown to increase the risk for complications (Atladóttir et al., 2010; Baud et al., 2008; Brown, 2011; Brown et al., 2000, 2004; Croen et al., 2005; Lee et al., 2015; Solek et al., 2018; Sørensen et al., 2009; Zerbo et al., 2015). A preterm birth mouse model activating maternal toll-like receptor (TLR)-4 results in an imbalance between adaptive and innate immune cells in the MFI (Arenas-Hernandez et al., 2015), potentially disrupting tolerance. Here, activating TLR-3 via the viral mimic poly(I:C) led to loss of PD-L1 (Figures 6E–6G). Given our short window of analysis, reduced expression is likely due to cell migration as opposed to protein-level changes. Simultaneously, we observed a reduction of Ly-6C_hi_ MPs (Figure 6H) and an expansion of conventional neutrophils within TIS (Figure 6I), mimicking acute inflammation and suggesting an abrupt disturbance in the equilibrium.

Our deep immune profiling of the MFI and PB enabled us to identify and noncanonical neutrophil and MP subpopulations linked to gestational immune remodeling. Our results suggest that innate immune cell diversity has been underappreciated, indicating the utility of high-dimensional approaches for tackling the complexities of the MFI. Overall, the analytical framework and emphasis on the dynamics of innate immune populations provide deeper resolution for pregnancy and its related pathologies.

### Limitations of the study

Our analysis treated the MFI as a unit, but we have yet to determine how the decidual and placental microenvironments uniquely shape maternal and fetal immune responses over gestation. The detection of fetal immune cells was challenging due to their limited numbers, and trogocytosis or non-specific antibody binding could have led to their mischaracterization. Our congenic mating strategy must be supplemented with additional methods to distinguish fetal immune cells. Future studies could include allogeneic pregnancies to address maternal-fetal tolerance and to characterize unique subpopulations in an allogeneic context. We hope that our work will serve as a resource and that the development, identity, and function of the cell phenotypes we described at the MFI will be studied further.

## STAR★Methods

### Key resources table

**Table:** 

REAGENT or RESOURCE	SOURCE	IDENTIFIER
**Antibodies**		

Biotin (Clone 1D4-C5)	BioLegend	Cat#409002
CD45 (Clone 30-F11)	BioLegend	Cat#103102
CD45.2 (Clone 104)	BioLegend	Cat#109802
Ter119 (Clone TER-119)	BioLegend	Cat#116202
B220 (Clone RA3-6B2)	BioLegend	Cat#103202
Ly-6G (Clone 1A8)	BioLegend	Cat#127602
CD11c (Clone N418)	BioLegend	Cat#117302
TCRβ (Clone H57-597)	BioLegend	Cat#109202
CD115 (Clone AFS98)	BioLegend	Cat#135502
CD69 (Clone H1.2F3)	BioLegend	Cat#104502
F4/80 (Clone BM8)	BioLegend	Cat#123102
CD3 (Clone 17A2)	BioLegend	Cat#100202
IgD (Clone 11-26c.2a)	BioLegend	Cat#405702
CD19 (Clone 6D5)	BioLegend	Cat#115502
CD25 (Clone 3C7)	BioLegend	Cat#101902
CD64 (Clone X54-5/7.1)	BioLegend	Cat#139302
CD80 (Clone 16-10A1)	BioLegend	Cat#104702
CD8 (Clone 53–6.7)	BioLegend	Cat#100702
CD11b (Clone M1/70)	BioLegend	Cat#101202
CD40 (Clone HM40-3)	BioLegend	Cat#102902
IgM (Clone RMM-1)	BioLegend	Cat#406502
CD117 (Clone 2B8)	BioLegend	Cat#105804
TCRγδ (Clone GL3)	BioLegend	Cat#118101
CTLA-4 (Clone UC10-4B9)	BioLegend	Cat#106302
Ly-6C (Clone HK1.4)	BioLegend	Cat#128002
CD194 (Clone 2G12)	BioLegend	Cat#131202
CD62L (Clone MEL-14)	BioLegend	Cat#104402
PD-L1 (Clone 10F.9G2)	BioLegend	Cat#124302
FcεRI-α (Clone MAR-1)	BioLegend	Cat#134302
CD335 (Clone 29A1.4)	BioLegend	Cat#137602
Siglec-F (Clone E50-2440)	BD Biosciences	Cat#552125
CD49b (Clone DX5)	BioLegend	Cat#108902
CD44 (Clone IM7)	BioLegend	Cat#103002
CD4 (Clone RM4-5)	BioLegend	Cat#100506
PD-1 (Clone 29F.1A12)	BioLegend	Cat#135202
MHC-II (Clone M5/114.15.2)	BioLegend	Cat#107602
CD86 (Clone GL-1)	BioLegend	Cat#105002
CD45.1 (Clone A20)	BioLegend	Cat#110702
FoxP3 (Clone FJK-16s) −158Gd	Fluidigm Sciences	Cat#3158003A
CD68 (Clone FA-11)	BioLegend	Cat#137002
RPS6 Ser235/Ser236 (Clone A17020B)	BioLegend	Cat#608602
CD31 (Clone MEC13.3)	BioLegend	Cat#102501

**Chemicals, peptides, and recombinant proteins**		

Poly(I:C) LMW	Invivogen	Cat#tlrl-picw
Iododeoxyuridine	Sigma-Aldrich	Cat #I7125
Cisplatin	Sigma Aldrich	Cat#P4394

**Critical commercial assays**		

K2EDTA evacuated blood collection tubes	Fisher Scientific	Cat#02-683-99A
RBC Lysis Buffer	BioLegend	Cat#420301
Accutase	Sigma Aldrich	Cat##SCR005
FOXP3 Transcription Factor Staining Buffer Set	eBioscience	Cat#00-5523-00
MaxPar X8 Antibody Labeling kit	Fluidigm Sciences	Cat#201300
TruStain FcX	BioLegend	Cat#101320
Cell-ID Intercalator-Ir	Fluidigm Sciences	Cat#201192
M.O.M. Immunodetection Kit	Vector Laboratories	Cat#BMK-2202

**Deposited data**		

Raw mass cytometry data	This study, Mendeley Data	https://doi.org/10.17632/45gz4r28s2.1

**Experimental models: Organisms/strains**		

Mouse: B6	The Jackson Laboratory	RRID: IMSR_JAX:000664
Mouse: B6.SJL-Ptprca Pepcb/BoyJ	The Jackson Laboratory	RRID: IMSR_JAX:002014

**Oligonucleotides**		

Sex genotyping forward primer: 5-CTGAAGCTTTTGGCTTTGAG-3′	This study	N/A
Sex genotyping reverse primer: 5-CCGCTGCCAAATTCTTTGC-3′	This study	N/A

**Software and algorithms**		

R	R Core Team, 2022	r-project.org
Python	N/A	python.org
ParkerICI/premessa	N/A	https://github.com/ParkerICI/premessa
Cydar	Lun et al., 2017	bioconductor.org/packages/release/bioc/html/cydar.html
gee	N/A	=CRAN.R-project.org/package = gee
limma	Ritchie et al., 2015	bioconductor.org/packages/release/bioc/html/limma.html
CellEngine	CellCarta, Montreal, Canada	cellengine.com
Single Cell Debarcoder	Zunder et al. 2015	github.com/nolanlab/single-cell-debarcoder
BioRender	BioRender	biorender.com
Scanpy	Wolf et al., 2018	scanpy.readthedocs.io
SciKit Learn	Pedregosa et al., 2011	scikit-learn.org
scikit-bio	The scikit-bio development team	scikit-bio.org
SciPy	Virtanen et al., 2020	scipy.org
statsmodels	Seabold and Perktold, 2010	statsmodels.org

**Other**		

Microarray gene expression of developing mouse decidua and placenta	Knox and Baker, 2008	GEO: GSE11220
Immune associated genes	Immport	immport.org/resources
Vascular development gene dataset	The Jackson Laboratory	GO:0001944

### Resource availability

#### Lead contact

Further information and request for resources and reagents should be directed to and will be fulfilled by the Lead Contact, Dr. Sean Bendall (bendall@stanford.edu).

#### Materials availability

The study did not generate new unique reagents.

### Experimental model and subject details

#### Animals

All mice were housed in an animal facility that is accredited by the Association for Assessment and Accreditation of Laboratory Animal Care International and maintained in specific pathogen-free conditions. Animal studies were conducted in accordance with National Institutes of Health guidelines for the humane use of animals and reviewed and approved by the Stanford Institutional Animal Care and Use Committee. Wild-type female and male C57BL/6J mice (RRID: IMSR_JAX:000664) between 6 and 8 weeks old were purchased from The Jackson Laboratory and housed at our facility. To differentiate between maternal and fetal cells, male C57BL/6 CD45.1 (B6.SJL-Ptprca Pepcb/BoyJ; RRID: IMSR_JAX:002014) mice between 7 and 8 weeks old were also purchased from The Jackson Laboratory. Animals were housed under standard 12-h light/dark cycles and fed with standard chow.

### Method details

#### Timed pregnancies and treatments

Timed pregnancies were generated by housing one or two naturally cycling females with a single male overnight. Mice were separated early the following morning and mating was assessed by the appearance of a copulation plug. The day of plug detection was classified as E0.5. For baseline studies, pregnant mice at embryonic days 10.5, 11.5, 12.5, 13.5, 14.5, 15.5, 16.5, 17.5, and 18.5 were injected intraperitoneally (i.p.) with saline 2–3 h before sacrifice. For perturbation studies, at E12.5 or 14.5, pregnant mice were treated with Poly(I:C) (Invivogen, Cat#tlrl-picw). Poly(I:C) was dosed at 18.5 mg/kg body weight, prepared at a concentration of 5 mg/mL in saline and injected i.p. 2–3 h before sacrifice. To assess proliferative activity, pregnant mice received one intraperitoneal injection of Iododeoxyuridine (IdU; Sigma-Aldrich, Cat#I7125). IdU was dissolved in saline after bringing pH to 10 with sodium hydroxide and incubating on a 37°C shaker. Once dissolved, pH was brought to 8.5 using hydrochloric acid and solution was filtered before injection. IdU was dosed at 100 mg/kg of body weight at a concentration of 10 mg/mL 2–3 h before sacrifice. S-phase takes 10-15 h to complete so this method only marks cells that are actively in S-phase as they reside in the respective compartments, so IdU positivity represents localized expansion.

#### Detection of endovascular immune cells

Mice were injected retro-orbitally at least 2 min before sacrifice with up to 5 μg biotin rat anti-mouse CD45 Ab (clone 30-F11; BioLegend, Cat#103104) in 60 μL saline. We analyzed samples by mass cytometry to determine successful anti-CD45 labeling in peripheral blood and its absence in matched cells from the lumbar lymph nodes (Figure S1F). Mice that showed significant Ab leakage into lymph nodes were excluded from downstream analysis.

#### Tissue preparation and cell isolation

After terminal anesthesia by ketamine and xylazine, peripheral blood was collected via cardiac puncture and transferred into K2EDTA evacuated blood collection tubes (Fisher Scientific, Cat#02-683-99A). Cells from the peripheral blood were subjected to RBC Lysis Buffer (BioLegend, Cat#420301) to remove red blood cells. Tissue was collected from each fetus for sex genotyping and stored at −20°C. Placentas (with decidua) were collected and minced with scissors in cold Accutase (Sigma Aldrich, Cat#SCR005) before transferring to a 37°C incubator shaker for enzymatic digestion as previously described (Arenas-Hernandez et al., 2015). Following digestion, samples were centrifuged at 1200 rpm for 2 min at RT. Aside from Figures S1C and S1K, placentas were not dissected into fetal and decidual (maternal) portions. Placentas were processed whole (including decidua) to reduce variability, preserve the capacity to compare samples across gestation, and maintain sample integrity. Complete decidual removal from the fetal spongiotrophoblast and trophoblast giant cells, while never guaranteed, was only effectively possible on a subset of embryonic days. Inconsistent decidual removal would prevent cross-gestational analysis of the placenta. Additionally, decidual removal required unavoidable physical pressure on the placenta, which resulted in endovascular leakage. Any leakage of this sort would prevent us from systematically profiling the endovascular compartment of the placenta. Samples were filtered through a 70 micron cell strainer (Falcon). All single-cell suspensions were quenched and washed with FACS buffer (PBS with 10% FCS and 5 mm EDTA) at 4°C. To label non-viable cells, all cells were resuspended at a 1:1 ratio with PBS with 5 mm EDTA and 100 μM cisplatin (Sigma Aldrich, Cat#P4394) for 1 min before quenching at a 1:1 ratio in FACS buffer. Cells were centrifuged at 1200 rpm for 5 min at 4°C and resuspended in FACS buffer and fixed for up to 1 h at RT using the FOXP3 Transcription Factor Staining Buffer Set (eBioscience, Cat#00-5523-00) at a cell density of 1 million cells per 500 μL final volume. Cells were then resuspended in FACS buffer and centrifuged at 1500 rpm for 5 min at 4°C. Samples were kept at 4°C during all steps of tissue harvest and cell isolation except enzymatic digestion, viability staining, and fixation. Cells were stored at −80°C until all samples were ready for staining.

#### Immunohistochemistry

Fresh placentas were embedded in OCT compound (Tissue-Tek) and flash frozen on dry ice before −80°C storage. Serial fresh frozen sections, cut at 5um, were post-fixed with 4% sucrose/4% PFA in PBS for 15 min at room temperature. After blocking endogenous peroxidase activity with BLOXALL Blocking solution (Vector Laboratories) for 30 min, sections were treated with Avidin/Biotin Blocking Kit (Vector Laboratories) to block endogenous biotin. M.O.M. Immunodetection Kit (Vector Laboratories) was applied for 1 h to block endogenous mouse IgG, followed by treatment with blocking buffer for 1 h. Sections were incubated at 4°C overnight with the appropriate biotinylated antibody: anti-mouse IgG isotype control (clone MOPC-173; BioLegend), anti-rat IgG isotype control (clone RTK4530; BioLegend), anti-mouse CD45.1 (clone A20; BioLegend), anti-rat CD45 (clone 30-F11; Biolegend), or anti-mouse CD31 (MEC13.3; BioLegend), all diluted 1:100 in 3% horse serum. The antibodies were detected with VECTASTAIN ABC-HRP Kit (Vector Laboratories) and revealed with DAB peroxidase substrate (Vector Laboratories). The sections were counterstained with hematoxylin (Sigma). Protocol details, buffers, and solutions can be found in published protocols.io (dx.doi.org/10.17504/protocols.io.bf6ajrae, dx.doi.org/10.17504/protocols.io.bhmej43e). Tissue slides scanned on NanoZoomer 2.0RS Digital slide scanner (Hamamatsu) and visualized on NDP.view2 Viewing software U12388-01 (Hamamatsu).

#### Sex genotyping

DNA was extracted from fetal body tissue for sex genotyping (Sigma Aldrich, Cat#XNAT). Primers used for sex genotyping PCR target the X-chromosome-specific gene Jarid1c and the Y-chromosome-specific gene Jarid1d (Forward primer: 5-CTGAAGCTTTTGGCTTTGAG-3′; Reverse primer: 5-CCGCTGCCAAATTCTTTGC-3′). Female samples exhibit a single band at 331 bp, whereas male samples have two bands at 302 and 331.

#### Mass cytometry antibody conjugation

Antibody conjugation was performed as previously described (Hartmann et al., 2019). Briefly, metal-isotope labeled antibodies were conjugated using the MaxPar X8 Antibody Labeling kit according to the manufacturer’s protocol (Fluidigm Sciences) or were purchased pre-conjugated (Fluidigm Sciences). To validate conjugation, the absorbance of the conjugated antibody was measured at 280 nm and the concentration was calculated, often resulting in over 60% recovery of antibody. Antibodies were titrated to determine the optimal staining concentration using primary mouse cells and/or mouse cell lines. For long-term storage at 4°C, antibodies were diluted in Antibody Stabilizer solution (Candor Bioscience GmbH, Cat#131-050) with 0.02% NaN3 (Merck Chemicals, Cat#106688) at 0.2 mg/mL. All mass cytometry antibodies and concentrations used in these studies can be found in Table S1.

#### Mass cytometry sample processing and data acquisition

Due to the number of samples that needed to be collected over a long period of time and the length of time CyTOF analysis requires, we needed to separate the samples into three large batches. Each batch was stained and analyzed at a separate time. Placentas were pooled separately by sex for each litter. Mass-tag cell barcoding was employed as previously described^3^ to pool same-organ samples for more efficient processing and measurement. Briefly, each sample was labeled with distinct combinations of six stable Pd isotopes in PBS with 0.02% saponin. Barcoded samples were washed with cell staining media (CSM; PBS with 0.5% BSA and 0.02% NaN3; Sigma Aldrich) and pooled into a single 5 mL round-bottom polystyrene test tube (Corning) for surface staining. Barcoded samples were suspended in TruStain FcX (BioLegend, Cat#101320) to prevent non-specific antibody binding and incubated on ice for 10 min prior to staining. Surface staining was performed in CSM in 500 μL total volume for 30 min at RT. Cells were washed in CSM and fixed (eBioscience, Cat#00-5523-00) for 10 min at RT. Cells were centrifuged at 1600 rpm for 5 min at 4°C and supernatant was aspirated after all washes. Cells were washed once in CSM and once in permeabilization buffer (eBioscience, Cat#00-5523-00). Cells were stained with intracellular antibodies in permeabilization buffer in 500 μL total volume for 30 min at RT. Cells were washed in CSM and stained with 1 mL DNA intercalation solution (1.6% PFA in low barium PBS with 0.02% saponin and 0.5 μM Cell-ID Intercalator-Ir; Fluidigm Sciences, Cat#201192) overnight at 4°C or until data acquisition, not exceeding 7 days. Before data acquisition, samples were washed once in CSM and twice in ddH2O. All samples were resuspended in 1x EQ Four Element Calibration Bead solution (Fluidigm Sciences, Cat#201078) with ddH2O at 1-2x10^6^ cells/mL and filtered through a cell strainer capped test tube (Falcon, Cat#352235) before being injected into a CyTOF2+ mass cytometer (Fluidigm Sciences) using the Super Sampler injection system (Victorian Airship and Scientific Apparatus).

#### Mass cytometry data processing

After cell acquisition, FCS files for each sample were bead normalized and concatenated with the ParkerICI/premessa package in R (https://github.com/ParkerICI/premessa). FCS files obtained from barcoded plates were then deconvoluted with the Single Cell Debarcoder application developed by Zunder et al. (Zunder et al., 2015). To correct for technical variation between CyTOF runs, we quantile normalized protein expression with the Cydar package in R (Lun et al., 2017). Each barcode plate included a splenocyte sample to which we normalized all samples across plates. These FCS files were then uploaded to CellEngine for gating (Figure S1A). All parameters except for time and cell length were displayed with an arcsinh cofactor 5 transformation. Events positive for intercalator-Ir were selected as having high DNA content. Cisplatin was then used to discriminate between live and dead cells. Staining with TER119 allowed exclusion of red blood cells from proceeding gates. Cells were then either gated for their expression of CD45.2+ single positive, deemed maternal immune, or CD45.2+CD45.1+ double-positive, deemed fetal immune (Figure S1A).

We established whether maternal immune cells were in placental tissue (TIS) or within the placenta’s endovasculature (EV) by setting a threshold based on the expression of retro-orbitally (R.O.) injected CD45-biotin in peripheral blood (PB) and lumbar lymph nodes (LN) (Figure S1I). With the arcsinh cofactor 5 transformation of cellular medians, we set the threshold to equal 3.5. In the placenta samples, any cells with median intensity equal to or higher than 3.5 were considered to be in EV. Any cells with R.O. CD45 that fell below the 3.5 threshold were considered to be in TIS. When we visualize the median intensity of R.O. CD45 across entire samples, we see a distinction in the expression of R.O. CD45 in EV vs. TIS (Figure S1M). We confirmed detection of EV immune cells by traditional gating as well (Figure S1L).

The maternal and fetal immune cell subsets identified in Figure 1, Figure 2 and 2 through dimensionality reduction and clustering were then confirmed using traditional gating methodology on CellEngine as seen in Figures S1L and S2D. We back-gated to ensure cells were not present in multiple gates. Furthermore, we identified the maternal mononuclear phagocyte (Figure 4A) and neutrophil (Figure 5A) subsets via traditional gating in Figures S4F and S5F, respectively. To ensure every cell was counted, we gated in a hierarchical manner as shown (Figures S4F and S5F).

Canonical maternal MP subsets were determined by their Ly-6C expression. Based on statistics of Ly-6C MP expression in peripheral blood, MPs were considered “classical” if their arcsinh cofactor 5 median expression was equal or higher than 4.5. Fiftieth percentile was 4.9, mean was 4.1, and standard deviation was equal to 2. “Intermediate” MPs were those with Ly-6C expression equal or higher than 3 but lower than 4.5 (25^th^ percentile was 2.7). Lastly, “non-classical” MPs had Ly-6C median intensity lower than 3.

### Quantification and statistical analysis

#### Dimensionality reduction and clustering

We used Scanpy’s Python based implementation (Wolf et al., 2018) to carry out dimensionality reduction via UMAP and clustering with the Leiden algorithm. These were carried out separately for maternal and fetal immune cells. Protein median intensities were first transformed with an inverse hyperbolic sine (arcsinh) with a cofactor of 5. We computed a UMAP neighborhood graph from baseline maternal immune data by randomly subsampling up to 500 single positive CD45.2+ cells from each mouse organ from embryonic day 10.5–18.5 and restricting local neighbors to 20. UMAP was based on the expression of the following lineage markers: Ly-6G, CD11c, TCRb, F4/80, CD3, IgD, IgM, CD19, CD8, CD11b, Ly-6C, FcεRI, Siglec-F, CD68, CD49b, CD4, and MHC-II. These same lineage markers were used to carry out Leiden clustering (Figure S1D).

Leiden produced 16 clusters, including 2 clusters that were excluded from downstream analysis due their doublet inclusion and broad expression of all lineage markers. With the remaining 14 clusters, we hierarchically clustered 2 B cell clusters, 2 neutrophil clusters, and 2 NK cell clusters into their broader cell types. Leiden also produced 5 mononuclear phagocyte (MP) clusters which had a range in expression of Ly-6C, MHC II, and CD11b. These clusters likely include macrophages (Mac), dendritic cells (DCs), monocytes, and monocytes differentiating into Mac or DCs. We decided to hierarchically cluster these cells because their over-clustering could be due to their low expression of other lineage markers, resulting in non-specific cellular distributions in the UMAP (Figures S1D and S1E). We acknowledge that heterogeneity also contributed to the generation of multiple clusters per cell type, but analyzing heterogeneity is best done by applying additional clustering markers that are relevant to and sufficiently expressed by these cells, as we did for MPs (Figure 4) and neutrophils (Figure 5). Finally, we identified one Leiden cluster that was composed of both eosinophils and basophils and spatially separated in the UMAP graph (Figures S1D, S1F). We set a threshold of arcsinh cofactor 5 transformed FcεRI median equal to 1 to split this single cluster into two distinct populations (Figure S1G). FcεRI is specific to basophils. Furthermore, we found very low levels of c-Kit in the “Basophils and Eosinophils” Leiden cluster (Figure S1F), suggesting the inclusion of mast cells. The c-Kit positive population was found to overlap with the location of NK cells in the UMAP graph. In the kernel density plot of c-Kit (Figure S1G), the levels of c-Kit positive cells were overwhelmed by the vastly c-Kit negative “Basophil and Eosinophil” population. Because we had such a low number of these mast cells, we decided to keep them in the heterogeneous eosinophil population.

We computed a UMAP neighborhood graph from baseline fetal immune data by subsampling up to 150 CD45.2+CD45.1+ cells from placentas on embryonic days 10.5, 12.5, 14.5, and 18.5, then applied the same Leiden setting used for the maternal immune cell analysis. Applying Leiden resulted in 13 clusters (Figure S2A). Seven of these clusters were classified as MPs with differential MHC-II, F4/80, and Ly-6C expression (Figure S2B), and were grouped into a single cluster. Similarly, two clusters were identified as neutrophils with differential MHC-II expression (Figure S2B) and were grouped into a single cluster. Finally, there was one cluster left unassigned because its marker expression was low for all lineage markers tested.

To further analyze maternal MP heterogeneity, we isolated maternal MPs and applied UMAP and Leiden using biologically relevant markers expressed in MPs: CD11c, F4/80, CD64, CD68, CD86, CD80, MHC-II, and cellularly incorporated IdU to track cell proliferation (Figure 4). The UMAP neighborhood graph was again restricted to 20 neighbors. Applying Leiden resulted in 11 MP clusters (Figure S4A). We found 3 Leiden clusters that were nearly identical based on their median protein expression (Figure S4C), so we grouped them into a single “Ly-6C_hi_” subset. Additionally, two MHC-II expressing clusters with negative CD11c were grouped into the “Presenting” subset. We removed an F4/80 high expressing cluster from downstream analysis because it spatially overlapped with several other clusters on the UMAP graph (Figures S4A and S4D). The “F4/80_hi_” cluster was only found in the peripheral blood.

To further analyze maternal neutrophil heterogeneity, we isolated maternal neutrophils and applied UMAP and Leiden using CD62L, MHC-II, CD80, CD40, PD-L1, and incorporated IdU to track cell proliferation (Figure 5). The UMAP neighborhood graph was restricted to 20 neighboring cells. Applying Leiden resulted in 11 clusters, which were then grouped based on the differential expression of CD11b, Ly-6C, Ly-6G, and CD44 in addition to the markers used for clustering (Figure S5). Four Leiden clusters were grouped under “conventional” and 3 under “presenting” neutrophils. One of the clusters Leiden identified was negative for all markers tested, including Ly-6G, so we removed it from further analysis.

We also removed from analysis technical artifacts and sample outliers if we observed metal isotope bleed-through, sample-specific clusters, and anomalous sample-driven clustering.

#### Scaled median

Protein median intensities were first transformed with an inverse hyperbolic sine (arcsinh) with a cofactor of 5. In Figure 5A, for only neutrophils, the arcsinh marker value (mean by mouse) underwent min-max normalization, meaning it was scaled with the following formula: (sample-min)/(max-min).

#### Linear discriminant analysis

We dimensionally reduced cell fractions of B cells, basophils, CD4 T cells, CD8 T cells, eosinophils, mononuclear phagocytes, NK cells, and neutrophils across the three compartments analyzed (TIS, EV, and PB) by implementing SciKit Learn’s (Pedregosa et al., 2011) Linear discriminant analysis (LDA). For Figure 1G, the compartments served as the three class labels, and the cell fractions within each compartment were the features. The LDA coefficients can be found in Figures S1I. In Figure 6B, we trained the LDA on saline samples (contour plots) and transformed the input cell frequencies from samples of Poly(I:C)-challenged mice. Poly(I:C) samples were then overlaid as points with their original class label. The LDA coefficients for this analysis be found in Figure S6B.

#### Bray-Curtis index of dissimilarity

Beta diversity is a ratio metric used in ecology to measure the degree of difference in species composition across communities or environments. We considered immune cells to be similar to a community of species. We used the scikit-bio.diversity beta subpackage and Bray-Curtis metric to measure the degree of difference between the three compartments analyzed (TIS, EV, and PB) using the cell abundance of B cells, basophils, CD4 T cells, CD8 T cells, eosinophils, mononuclear phagocytes, NK cells, and neutrophils (Figure 1, Figure 6H and 6C).

#### Microarray data

Publicly available mouse placenta and decidua microarray data (Knox and Baker, 2008) was analyzed for immune and vascular development genes (Figure 3B). The immune gene dataset was obtained from Immport (https://www.immport.org/resources), and the vascular development gene dataset was obtained from Jackson Laboratories (GO:0001944). We implemented R to determine differentially expressed genes with the aid of the limma package (Ritchie et al., 2015) and focused on genes that changed between E8.5 and 15.5. The full dataset of immune and vascular associated genes we found to be differentially expressed between E8.5 and 15.5 can be found in the supplemental files.

#### Linear regression

We used SciKit-Learn’s implementation of linear regression to determine if the frequency of B cells, basophils, CD4 T cells, CD8 T cells, eosinophils, mononuclear phagocytes, NK cells, and neutrophils demonstrated temporal organization across the three compartments analyzed (Figure S3B). The features we used were the fractions of the given cell types (calculated out of all immune cells in the compartment) and our target values were embryonic days. Linear regression coefficients are shown in Figure S3C. We used SciKit-Learn’s cross validation feature to determine the Ridge regression score function (R^2^), allowing us to evaluate our model’s embryonic day prediction based on immune cell composition. The R^2^ when using all cells as features is found in S3F. We additionally applied linear regression only using the frequency of MPs and neutrophils across compartments recapitulating a similar pattern in accuracy scores when comparing compartments (Figure 3E). We carried out linear regression in the endovascular (EV) compartment in early (E10.5 to 13.5) and late (E14.5 to 18.5) periods of gestation using all cell types (Figure S3D). The coefficients of this regression can be found in Figure S3E. The R^2^ scores using all cell types as features for EV gestational period are shown in Figure S3F, while the score for using only MPs and neutrophils can be found in Figure 3E.

#### Generalized estimating equations

To estimate the effect of gestational day across placental compartments (Figure 3, Figure 4, Figure 5, 4, and 5), linear regression coefficient and standard error estimates were calculated using the Generalized Estimating Equations (GEE) framework (Liang and Zeger, 1986). Separate regressions were run depending on cell type, cell function, or protein marker. In each regression, the main effects of gestational day and compartment were included along with the interaction with day and day-squared where appropriate. Using a cluster size of 1 and an independent correlation structure, this approach was equivalent to using heteroskedastic-robust standard errors where the variance of error terms are not identically distributed, but are instead estimated using the squared residual from the individual observations (Eicker, 1967; Huber, 1967; White, 1980).

For comparing average median protein intensity between compartments within a given cell type, the same regression models were run using only the main effects of the EV and TIS compartments without gestational day producing coefficient estimates which compared each compartment against PB. Similar results can be accomplished via a one sided t test.

All regressions were run in R using the gee package. Controlling for heteroskedasticity was justified by visual examination of the residuals plotted against the fitted values in each regression category.

#### Statistics

Statistical tests comparing means of two independent samples were performed with the assistance of the SciPy (Virtanen et al., 2020) statistics module using T tests. To calculate p values for two ratio comparisons (Figures 6H and 6I), we applied SciPy’s T test for two independent samples from descriptive statistics. To calculate the standard deviation of the ratio of two independent variables (Figures 6H and 6I), we took the square root of the variance, which was calculated using the Taylor Series: V(X/Y) = E(X2/Y2) - [E(X/Y)]2 = E(X2)·E[(1/Y)2] - [E(X)·E(1/Y)]2. When indicated, we adjusted p values with the Bonferroni method using the Python module statsmodels (Seabold and Perktold, 2010; Figure 3F).

One-way analysis of variance (ANOVA) was used when comparing three or more means of independent samples. Pingouin (Vallat, 2018) was used to determine homogeneity of variances, apply classic ANOVA if the groups being compared had equal variances or use the Welch ANOVA for groups with unequal variances. Additionally, the Tukey-HSD post-hoc test was used following a classic ANOVA, and the Games-Howell test was used for samples with unequal variances.

Significant p values are shown as follows on figures: ^∗^p ≤ 0.05, ^∗∗^p ≤ 0.01, ^∗∗∗^p ≤ 0.001, ^∗∗∗∗^p ≤ 0.0001.

## Data Availability

All relevant single-cell mass cytometry data have been deposited to Mendeley Data and are publicly available as of the date of publication. Relevant DOI is listed on the key resources table. This paper does not report original code. Any additional information required to reanalyze the data reported in this paper is available from the lead contact upon request.

## Supplemental information

Document S1. Figures S1–S6, Tables S1, and S4–S10

Table S2. Microarray immune-associated genes. Related to Figure 3B The table shows the expression of immune-associated genes that are differentially expressed between E8.5 and 15.5 in the publicly available placental microarray data from Knox and Baker (2008).

Table S3. Microarray vascular-associated genes. Related to Figure 3B The table shows the expression of vascular-associated genes that are differentially expressed between E8.5 and 15.5 in the publicly available placental microarray data from Knox and Baker (2008).

Document S2. Article plus supplemental information
