# Supplementary material for: Gestationally dependent immune organization at the maternal-fetal interface
Source: Cell Rep. 2022 Nov 15;41(7):111651. doi: 10.1016/j.celrep.2022.111651 (PMC9681661; doi:10.1016/j.celrep.2022.111651)
Supplement: Document S1. Figures S1–S6, Tables S1, and S4–S10 [file mmc1.pdf]

Cell Reports, Volume *41*

Supplemental information

Gestationally dependent immune organization at the maternal-fetal interface

Amber R. Moore, Nora Vivanco Gonzalez, Katherine A. Plummer, Olivia R. Mitchel, Harleen Kaur, Moises Rivera, Brian Collica, Mako Goldston, Ferda Filiz, Michael Angelo, Theo D. Palmer, and Sean C. Bendall

SUPPLEMENTAL TABLES and FIGURES

Table S1. A summary of antibodies used for our mass cytometry analysis. Related to Figure 1. The table shows the antibody target, antibody clone, the element and isotope the antibody was conjugated to, the final concentration of the metal-conjugated antibody used for protein detection, and the vendor from whom the antibodies were purchased.

Antibody target	Clone	Element	Mass	Concentration (ug/ml)	Vendor
**Biotin**	1D4-C5	In	113	2	BioLegend
**CD45**	30-F11	see footnote			BioLegend
**CD45.2**	104	In	115	4	BioLegend
**Ter119**	TER-119	La	139	2	BioLegend
**B220**	RA3-6B2	Ce	140	4	BioLegend
**Ly-6G**	1A8	Pr	141	2	BioLegend
**CD11c**	N418	Nd	142	1	BioLegend
**TCRβ**	H57-597	Nd	143	1	BioLegend
**CD115 (CSF-1R)**	AFS98	Nd	144	2	Fluidigm Sciences
**CD69**	H1.2F3	Nd	145	1	BioLegend
**F4/80**	BM8	Nd	146	1	BioLegend
**CD3**	17A2	Sm	147	2	BioLegend
**IgD**	11-26c.2a	Nd	148	2	BioLegend
**CD19**	6D5	Sm	149	1	BioLegend
**CD25**	3C7	Nd	150	4	Fluidigm Sciences
**CD64**	X54-5/7.1	Eu	151	4	BioLegend
**CD80**	16-10A1	Sm	152	2	BioLegend
**CD8**	53-6.7	Eu	153	1	BioLegend
**CD11b**	M1/70	Sm	154	1	BioLegend
**CD40**	HM40-3	Gd	155	2	BioLegend
**IgM**	RMM-1	Gd	156	1	BioLegend
**CD117 (c-Kit)**	2B8	Gd	157	0.5	BioLegend
**TCRγδ**	GL3	Tb	159	2	BioLegend
**CTLA-4 (CD152)**	UC10-4B9	Dy	161	2	BioLegend
**Ly-6C**	HK1.4	Dy	162	2	BioLegend
**CD194 (CCR4)**	2G12	Dy	163	2	BioLegend
**CD62L**	MEL-14	Dy	164	2	BioLegend
**PD-L1 (CD274)**	10F.9G2	Ho	165	2	BioLegend
**FcεRI-α**	MAR-1	Er	166	0.5	BioLegend
**CD335 (Nkp46)**	29A1.4	Er	167	2	BioLegend
**Siglec-F**	E50-2440	Er	168	1	BDBiosciences
**CD49b**	DX5	Er	170	2	BioLegend
**CD44**	IM7	Yb	171	1	BioLegend
**CD4**	RM4-5	Yb	172	1	BioLegend
**PD-1 (CD279)**	29F.1A12	Yb	173	2	BioLegend
**MHC-II**	M5/114.15.2	Yb	174	1	BioLegend
**CD86**	GL-1	Lu	175	2	BioLegend
**CD45.1**	A20	Yb	176	2	BioLegend
**FoxP3**	FJK-16s	Gd	158	4	Fluidigm Sciences
**CD68**	FA-11	Tm	169	2	BioLegend

**prpS6 Ser235/Ser236**	A17020B	Bi	209	2	BioLegend

Footnote: the anti-CD45 antibody administered retro-orbitally is detected by the anti-biotin antibody (isotope In113)

Table S4. Generalized Estimating Equations coefficients and statistics for regressions of immune cell fractions over embryonic days. Related to Figure 3C. Generalized Estimating Equations (GEE) was used to fit linear models and compare compartments within each cell type. PB was used as the reference.

Coefficient	Estimate	Naive.S.E.	Naive.z	Robust.S.E.	Robust.z	P value	Cell type
**Intercept**	2.66E-01	6.48E-02	4.11E+00	5.50E-02	4.84E+00	6.41E-07	B cells
**Day**	3.93E-03	4.38E-03	8.96E-01	3.59E-03	1.09E+00	1.37E-01	B cells
**EV**	-2.14E-01	9.17E-02	-2.33E+00	6.63E-02	-3.22E+00	6.34E-04	B cells
**TIS**	-2.26E-01	9.17E-02	-2.46E+00	8.83E-02	-2.56E+00	5.29E-03	B cells
**Day:EV**	3.81E-03	6.19E-03	6.15E-01	4.51E-03	8.44E-01	1.99E-01	B cells
**Day:TIS**	-1.45E-03	6.19E-03	-2.35E-01	6.21E-03	-2.34E-01	4.07E-01	B cells
**Intercept**	2.55E-02	1.68E-02	1.52E+00	1.68E-02	1.52E+00	6.42E-02	Basophils
**Day**	-7.89E-04	1.14E-03	-6.91E-01	1.03E-03	-7.63E-01	2.23E-01	Basophils
**EV**	-9.31E-03	2.55E-02	-3.65E-01	1.72E-02	-5.42E-01	2.94E-01	Basophils
**TIS**	2.67E-03	2.37E-02	1.13E-01	2.46E-02	1.08E-01	4.57E-01	Basophils
**Day:EV**	4.37E-05	1.72E-03	2.55E-02	1.06E-03	4.13E-02	4.84E-01	Basophils
**Day:TIS**	1.49E-03	1.60E-03	9.28E-01	1.64E-03	9.07E-01	1.82E-01	Basophils
**Intercept**	8.86E-02	2.77E-02	3.20E+00	4.79E-02	1.85E+00	3.23E-02	CD4+ T cells
**Day**	-9.08E-04	1.87E-03	-4.86E-01	3.04E-03	-2.99E-01	3.82E-01	CD4+ T cells
**EV**	-1.20E-01	3.91E-02	-3.06E+00	4.98E-02	-2.40E+00	8.14E-03	CD4+ T cells
**TIS**	-7.16E-02	3.91E-02	-1.83E+00	5.24E-02	-1.37E+00	8.58E-02	CD4+ T cells
**Day:EV**	5.30E-03	2.64E-03	2.01E+00	3.21E-03	1.65E+00	4.93E-02	CD4+ T cells
**Day:TIS**	1.48E-03	2.64E-03	5.61E-01	3.37E-03	4.39E-01	3.30E-01	CD4+ T cells
**Intercept**	8.33E-02	2.07E-02	4.03E+00	3.57E-02	2.33E+00	9.84E-03	CD8+ T cells
**Day**	-1.14E-03	1.40E-03	-8.16E-01	2.30E-03	-4.96E-01	3.10E-01	CD8+ T cells
**EV**	-1.09E-01	2.92E-02	-3.72E+00	3.66E-02	-2.97E+00	1.51E-03	CD8+ T cells
**TIS**	-6.50E-02	2.92E-02	-2.23E+00	4.04E-02	-1.61E+00	5.37E-02	CD8+ T cells
**Day:EV**	4.48E-03	1.97E-03	2.27E+00	2.36E-03	1.90E+00	2.85E-02	CD8+ T cells
**Day:TIS**	1.18E-03	1.97E-03	5.99E-01	2.67E-03	4.42E-01	3.29E-01	CD8+ T cells
**Intercept**	3.17E-02	1.36E-02	2.33E+00	1.95E-02	1.62E+00	5.24E-02	Eosinophils
**Day**	-1.60E-03	9.22E-04	-1.74E+00	1.17E-03	-1.36E+00	8.64E-02	Eosinophils
**EV**	-4.06E-03	1.91E-02	-2.12E-01	2.26E-02	-1.80E-01	4.29E-01	Eosinophils

**TIS**	8.14E-03	1.91E-02	4.25E-01	2.31E-02	3.53E-01	3.62E-01	Eosinophils
**Day:EV**	5.33E-04	1.30E-03	4.11E-01	1.36E-03	3.93E-01	3.47E-01	Eosinophils
**Day:TIS**	5.59E-04	1.30E-03	4.31E-01	1.46E-03	3.84E-01	3.51E-01	Eosinophils
**Intercept**	1.80E-01	7.10E-02	2.53E+00	4.68E-02	3.85E+00	6.00E-05	Monocytes
**Day**	-4.48E-03	4.80E-03	-9.34E-01	3.04E-03	-1.47E+00	7.06E-02	Monocytes
**EV**	9.79E-01	1.00E-01	9.75E+00	6.72E-02	1.46E+01	2.02E-48	Monocytes
**TIS**	3.72E-01	1.00E-01	3.70E+00	8.18E-02	4.54E+00	2.80E-06	Monocytes
**Day:EV**	-3.91E-02	6.78E-03	-5.77E+00	4.63E-03	-8.44E+00	1.55E-17	Monocytes
**Day:TIS**	-6.67E-03	6.78E-03	-9.83E-01	5.77E-03	-1.15E+00	1.24E-01	Monocytes
**Intercept**	2.46E-01	5.42E-02	4.54E+00	7.94E-02	3.10E+00	9.77E-04	NK cells
**Day**	3.90E-04	3.66E-03	1.06E-01	5.30E-03	7.36E-02	4.71E-01	NK cells
**EV**	-2.46E-01	7.67E-02	-3.21E+00	8.06E-02	-3.05E+00	1.14E-03	NK cells
**TIS**	-8.02E-02	7.67E-02	-1.05E+00	8.63E-02	-9.29E-01	1.76E-01	NK cells
**Day:EV**	2.63E-03	5.18E-03	5.08E-01	5.38E-03	4.90E-01	3.12E-01	NK cells
**Day:TIS**	-5.82E-03	5.18E-03	-1.12E+00	5.77E-03	-1.01E+00	1.56E-01	NK cells
**Intercept**	7.59E-02	8.43E-02	9.01E-01	3.75E-02	2.02E+00	2.15E-02	Neutrophils
**Day**	4.86E-03	5.69E-03	8.54E-01	2.58E-03	1.88E+00	2.98E-02	Neutrophils
**EV**	-2.70E-01	1.19E-01	-2.26E+00	6.67E-02	-4.04E+00	2.63E-05	Neutrophils
**TIS**	6.28E-02	1.19E-01	5.26E-01	9.32E-02	6.73E-01	2.50E-01	Neutrophils
**Day:EV**	2.18E-02	8.05E-03	2.71E+00	4.67E-03	4.67E+00	1.53E-06	Neutrophils
**Day:TIS**	8.97E-03	8.05E-03	1.11E+00	6.77E-03	1.32E+00	9.26E-02	Neutrophils

Table S5. Generalized Estimating Equations coefficients and statistics for regressions of protein medians in MP and neutrophils over embryonic days. Related to Figure 3G. GEE was used to fit linear models per protein and cell type to compare compartments. PB was used as the reference.

Coefficient	Estimate	Naive.S.E.	Naive.z	Robust.S.E.	Robust.z	P value	Cell/protein
**Intercept**	7.92E-01	5.71E-01	1.39E+00	3.58E-01	2.21E+00	1.34E-02	MP/PD-L1
**Day**	-3.01E-02	3.85E-02	-7.80E-01	2.28E-02	-1.32E+00	9.32E-02	MP/PD-L1
**EV**	1.84E+00	8.07E-01	2.28E+00	9.41E-01	1.95E+00	2.54E-02	MP/PD-L1
**TIS**	3.65E+00	8.07E-01	4.52E+00	6.28E-01	5.81E+00	3.20E-09	MP/PD-L1
**Day:EV**	-1.17E-01	5.45E-02	-2.15E+00	5.85E-02	-2.01E+00	2.25E-02	MP/PD-L1
**Day:TIS**	-1.76E-01	5.45E-02	-3.22E+00	4.13E-02	-4.26E+00	1.03E-05	MP/PD-L1
**Intercept**	3.49E-01	1.45E-01	2.41E+00	1.19E-01	2.94E+00	1.63E-03	Neutrophils/PD-L1
**Day**	-1.28E-02	9.78E-03	-1.30E+00	7.75E-03	-1.65E+00	5.00E-02	Neutrophils/PD-L1
**EV**	-7.80E-02	2.05E-01	-3.81E-01	1.77E-01	-4.40E-01	3.30E-01	Neutrophils/PD-L1
**TIS**	3.47E-01	2.05E-01	1.69E+00	1.93E-01	1.80E+00	3.59E-02	Neutrophils/PD-L1
**Day:EV**	3.70E-03	1.38E-02	2.68E-01	1.08E-02	3.42E-01	3.66E-01	Neutrophils/PD-L1
**Day:TIS**	-1.33E-02	1.38E-02	-9.63E-01	1.27E-02	-1.05E+00	1.47E-01	Neutrophils/PD-L1
**Intercept**	4.55E+00	6.85E-01	6.64E+00	9.90E-01	4.59E+00	2.20E-06	MP/Ly-6C
**Day**	8.76E-03	4.63E-02	1.89E-01	6.59E-02	1.33E-01	4.47E-01	MP/Ly-6C
**EV**	-9.56E-01	9.69E-01	-9.87E-01	9.73E-01	-9.83E-01	1.63E-01	MP/Ly-6C
**TIS**	1.12E+00	9.69E-01	1.16E+00	9.55E-01	1.18E+00	1.20E-01	MP/Ly-6C
**Day:EV**	7.25E-02	6.54E-02	1.11E+00	6.62E-02	1.10E+00	1.36E-01	MP/Ly-6C
**Day:TIS**	-1.10E-01	6.54E-02	-1.69E+00	6.93E-02	-1.59E+00	5.55E-02	MP/Ly-6C
**Intercept**	2.97E+00	2.67E-01	1.11E+01	3.89E-01	7.64E+00	1.12E-14	Neutrophils/Ly-6G
**Day**	-4.26E-03	1.80E-02	-2.36E-01	2.50E-02	-1.70E-01	4.32E-01	Neutrophils/Ly-6G
**EV**	-1.17E+00	3.77E-01	-3.09E+00	3.35E-01	-3.48E+00	2.54E-04	Neutrophils/Ly-6G
**TIS**	-1.31E+00	3.77E-01	-3.47E+00	3.42E-01	-3.83E+00	6.36E-05	Neutrophils/Ly-6G
**Day:EV**	6.39E-02	2.55E-02	2.51E+00	2.17E-02	2.95E+00	1.58E-03	Neutrophils/Ly-6G
**Day:TIS**	6.30E-02	2.55E-02	2.47E+00	2.31E-02	2.72E+00	3.22E-03	Neutrophils/Ly-6G

Table S6. Generalized Estimating Equations coefficients and statistics for regressions of immune fractions in MP subsets over embryonic days. Related to Figure 4E and S4G. GEE was used to fit linear models and compare fraction of immune cells in compartments within each MP subset. PB was used as the reference.

Coefficient	Estimate	Naive.S.E.	Naive.z	Robust.S.E.	Robust.z	P value	Cell type
**Intercept**	6.69E-02	3.97E-02	1.68E+00	3.28E-02	2.04E+00	2.06E-02	Ly-6Chi
**Day**	-4.63E-04	2.68E-03	-1.73E-01	2.04E-03	-2.27E-01	4.10E-01	Ly-6Chi
**EV**	1.02E-01	5.61E-02	1.82E+00	5.88E-02	1.74E+00	4.11E-02	Ly-6Chi
**TIS**	3.41E-02	5.61E-02	6.08E-01	5.01E-02	6.81E-01	2.48E-01	Ly-6Chi
**Day:EV**	3.34E-03	3.79E-03	8.82E-01	3.86E-03	8.66E-01	1.93E-01	Ly-6Chi
**Day:TIS**	4.79E-04	3.79E-03	1.26E-01	3.30E-03	1.45E-01	4.42E-01	Ly-6Chi
**Intercept**	3.98E-02	1.84E-02	2.17E+00	1.09E-02	3.64E+00	1.34E-04	Patrolling
**Day**	-1.29E-03	1.24E-03	-1.04E+00	7.14E-04	-1.81E+00	3.55E-02	Patrolling
**EV**	1.73E-01	2.60E-02	6.65E+00	2.96E-02	5.85E+00	2.50E-09	Patrolling
**TIS**	9.81E-03	2.60E-02	3.77E-01	1.60E-02	6.13E-01	2.70E-01	Patrolling
**Day:EV**	-9.79E-03	1.76E-03	-5.58E+00	1.91E-03	-5.11E+00	1.57E-07	Patrolling
**Day:TIS**	-5.33E-04	1.76E-03	-3.03E-01	1.05E-03	-5.05E-01	3.07E-01	Patrolling
**Intercept**	1.05E-02	3.19E-02	3.30E-01	1.43E-03	7.37E+00	8.71E-14	Phagocytic
**Day**	-4.30E-04	2.11E-03	-2.04E-01	9.19E-05	-4.68E+00	1.42E-06	Phagocytic
**EV**	5.79E-02	3.95E-02	1.47E+00	2.16E-02	2.68E+00	3.63E-03	Phagocytic
**TIS**	1.55E-02	3.95E-02	3.92E-01	2.86E-02	5.41E-01	2.94E-01	Phagocytic
**Day:EV**	-2.57E-03	2.63E-03	-9.76E-01	1.35E-03	-1.91E+00	2.84E-02	Phagocytic
**Day:TIS**	3.21E-03	2.63E-03	1.22E+00	2.02E-03	1.59E+00	5.62E-02	Phagocytic
**Intercept**	-2.30E-04	7.59E-02	-3.03E-03	1.51E-03	-1.53E-01	4.39E-01	MoDC
**Day**	2.13E-04	5.73E-03	3.72E-02	1.31E-04	1.63E+00	5.20E-02	MoDC

**EV**	2.23E-01	8.31E-02	2.68E+00	5.44E-02	4.10E+00	2.06E-05	MoDC
**TIS**	1.83E-01	8.31E-02	2.20E+00	2.18E-02	8.36E+00	3.01E-17	MoDC
**Day:EV**	-1.24E-02	6.17E-03	-2.01E+00	3.39E-03	-3.66E+00	1.29E-04	MoDC
**Day:TIS**	-8.41E-03	6.17E-03	-1.36E+00	1.49E-03	-5.63E+00	8.93E-09	MoDC
**Intercept**	7.84E-03	1.12E-02	7.01E-01	4.87E-03	1.61E+00	5.36E-02	DC
**Day**	-1.98E-04	7.80E-04	-2.54E-01	3.24E-04	-6.11E-01	2.71E-01	DC
**EV**	2.42E-02	1.53E-02	1.58E+00	1.32E-02	1.84E+00	3.32E-02	DC
**TIS**	8.80E-03	1.53E-02	5.76E-01	9.62E-03	9.15E-01	1.80E-01	DC
**Day:EV**	2.77E-05	1.05E-03	2.64E-02	8.49E-04	3.26E-02	4.87E-01	DC
**Day:TIS**	4.00E-04	1.05E-03	3.81E-01	6.84E-04	5.84E-01	2.80E-01	DC
**Intercept**	6.01E-02	2.68E-02	2.25E+00	1.00E-02	5.99E+00	1.07E-09	Presenting
**Day**	-2.78E-03	1.81E-03	-1.54E+00	6.34E-04	-4.39E+00	5.73E-06	Presenting
**EV**	2.60E-01	3.78E-02	6.87E+00	3.30E-02	7.86E+00	1.86E-15	Presenting
**TIS**	1.71E-02	3.78E-02	4.52E-01	2.54E-02	6.73E-01	2.50E-01	Presenting
**Day:EV**	-9.92E-03	2.56E-03	-3.88E+00	2.26E-03	-4.39E+00	5.54E-06	Presenting
**Day:TIS**	1.48E-03	2.56E-03	5.81E-01	1.81E-03	8.19E-01	2.06E-01	Presenting
**Intercept**	4.58E-03	1.84E-02	2.49E-01	1.94E-03	2.36E+00	9.05E-03	Proliferating
**Day**	-1.58E-04	1.29E-03	-1.23E-01	1.25E-04	-1.27E+00	1.02E-01	Proliferating
**EV**	4.80E-02	2.04E-02	2.35E+00	7.05E-03	6.81E+00	4.75E-12	Proliferating
**TIS**	5.72E-02	2.03E-02	2.82E+00	1.08E-02	5.29E+00	6.20E-08	Proliferating
**Day:EV**	-2.86E-03	1.43E-03	-2.00E+00	4.55E-04	-6.28E+00	1.71E-10	Proliferating
**Day:TIS**	-3.11E-03	1.42E-03	-2.19E+00	6.85E-04	-4.54E+00	2.77E-06	Proliferating
**Intercept**	5.55E-03	1.11E-01	4.99E-02	9.78E-02	5.68E-02	4.77E-01	F4/80hi
**Day**	5.95E-03	7.44E-03	8.00E-01	7.33E-03	8.12E-01	2.08E-01	F4/80hi

.

Table S7. Generalized Estimating Equations coefficients and statistics for regressions of PD-L1+ MP fractions in PD-L1+ MP subsets over embryonic days. Related to Figure 4F. GEE was used to fit linear models and compare fraction of PD-L1+ MPs in compartments within each PD-L1+ MP subset. PB was used as the reference.

Coefficient	Estimate	Naive.S.E.	Naive.z	Robust.S.E.	Robust.z	P value	Cell type
**Intercept**	8.05E-01	1.41E-01	5.72E+00	1.30E-01	6.19E+00	3.06E-10	Patrolling
**Day**	5.23E-03	9.50E-03	5.51E-01	8.42E-03	6.21E-01	2.67E-01	Patrolling
**EV**	-4.08E-01	1.99E-01	-2.05E+00	2.13E-01	-1.92E+00	2.75E-02	Patrolling
**TIS**	-6.24E-01	1.99E-01	-3.14E+00	1.60E-01	-3.91E+00	4.64E-05	Patrolling
**Day:EV**	-2.53E-03	1.34E-02	-1.89E-01	1.38E-02	-1.84E-01	4.27E-01	Patrolling
**Day:TIS**	-6.35E-03	1.34E-02	-4.73E-01	1.07E-02	-5.93E-01	2.77E-01	Patrolling
**Intercept**	3.28E-01	1.33E-01	2.46E+00	6.66E-02	4.92E+00	4.32E-07	Phagocytic
**Day**	-1.14E-02	8.81E-03	-1.30E+00	4.31E-03	-2.65E+00	4.00E-03	Phagocytic
**EV**	-3.40E-01	1.65E-01	-2.06E+00	9.87E-02	-3.45E+00	2.82E-04	Phagocytic
**TIS**	-4.65E-01	1.65E-01	-2.82E+00	1.24E-01	-3.75E+00	8.97E-05	Phagocytic
**Day:EV**	2.80E-02	1.10E-02	2.55E+00	6.41E-03	4.37E+00	6.26E-06	Phagocytic
**Day:TIS**	5.12E-02	1.10E-02	4.66E+00	8.86E-03	5.78E+00	3.69E-09	Phagocytic
**Intercept**	-1.44E-01	2.26E-01	-6.35E-01	9.58E-02	-1.50E+00	6.67E-02	MoDC
**Day**	1.80E-02	1.71E-02	1.05E+00	7.76E-03	2.31E+00	1.03E-02	MoDC
**EV**	7.60E-01	2.48E-01	3.07E+00	1.62E-01	4.68E+00	1.44E-06	MoDC
**TIS**	1.10E+00	2.48E-01	4.44E+00	1.20E-01	9.19E+00	2.02E-20	MoDC
**Day:EV**	-3.73E-02	1.84E-02	-2.03E+00	1.18E-02	-3.17E+00	7.63E-04	MoDC
**Day:TIS**	-5.66E-02	1.84E-02	-3.08E+00	9.27E-03	-6.11E+00	5.04E-10	MoDC

Table S8. Generalized Estimating Equations coefficients and statistics for regressions of fractions out of MPs in MP subsets over embryonic days. Related to Figure 4 and S4H. GEE was used to fit linear models and compare fraction out of MPs in compartments within each MP subset. PB was used as the reference.

Coefficient	Estimate	Naive.S.E.	Naive.z	Robust.S.E.	Robust.z	P value	Cell type
**Intercept**	2.12E-01	1.03E-01	2.05E+00	1.44E-01	1.47E+00	7.09E-02	Ly-6Chi
**Day**	2.07E-02	6.98E-03	2.96E+00	9.84E-03	2.10E+00	1.77E-02	Ly-6Chi
**EV**	-3.02E-01	1.46E-01	-2.07E+00	1.62E-01	-1.86E+00	3.13E-02	Ly-6Chi
**TIS**	-7.74E-02	1.46E-01	-5.29E-01	1.70E-01	-4.54E-01	3.25E-01	Ly-6Chi
**Day:EV**	1.58E-02	9.88E-03	1.60E+00	1.11E-02	1.42E+00	7.85E-02	Ly-6Chi
**Day:TIS**	-9.38E-03	9.88E-03	-9.50E-01	1.17E-02	-8.05E-01	2.10E-01	Ly-6Chi
**Intercept**	2.34E-01	6.50E-02	3.59E+00	8.53E-02	2.74E+00	3.09E-03	Patrolling
**Day**	-3.19E-03	4.39E-03	-7.26E-01	5.89E-03	-5.42E-01	2.94E-01	Patrolling
**EV**	3.10E-02	9.20E-02	3.37E-01	9.53E-02	3.25E-01	3.73E-01	Patrolling
**TIS**	-1.38E-01	9.20E-02	-1.50E+00	9.24E-02	-1.50E+00	6.73E-02	Patrolling
**Day:EV**	-8.52E-03	6.21E-03	-1.37E+00	6.53E-03	-1.30E+00	9.61E-02	Patrolling
**Day:TIS**	1.39E-03	6.21E-03	2.24E-01	6.39E-03	2.18E-01	4.14E-01	Patrolling
**Intercept**	6.55E-02	6.59E-02	9.94E-01	2.01E-02	3.25E+00	5.72E-04	Phagocytic
**Day**	-2.10E-03	4.36E-03	-4.81E-01	1.39E-03	-1.50E+00	6.62E-02	Phagocytic
**EV**	7.10E-03	8.16E-02	8.71E-02	3.76E-02	1.89E-01	4.25E-01	Phagocytic
**TIS**	-8.16E-02	8.16E-02	-1.00E+00	7.37E-02	-1.11E+00	1.34E-01	Phagocytic
**Day:EV**	4.18E-04	5.43E-03	7.70E-02	2.46E-03	1.70E-01	4.33E-01	Phagocytic
**Day:TIS**	1.65E-02	5.43E-03	3.04E+00	5.35E-03	3.09E+00	1.01E-03	Phagocytic
**Intercept**	-1.11E-02	1.24E-01	-9.00E-02	1.34E-02	-8.29E-01	2.04E-01	MoDC

**Day**	2.49E-03	9.34E-03	2.67E-01	1.19E-03	2.08E+00	1.85E-02	MoDC
**EV**	3.15E-01	1.35E-01	2.32E+00	7.60E-02	4.14E+00	1.74E-05	MoDC
**TIS**	4.66E-01	1.35E-01	3.44E+00	5.76E-02	8.09E+00	2.94E-16	MoDC
**Day:EV**	-1.78E-02	1.01E-02	-1.77E+00	4.83E-03	-3.69E+00	1.11E-04	MoDC
**Day:TIS**	-2.17E-02	1.01E-02	-2.16E+00	3.80E-03	-5.72E+00	5.30E-09	MoDC
**Intercept**	5.21E-02	4.09E-02	1.28E+00	7.02E-02	7.42E-01	2.29E-01	DC
**Day**	1.50E-04	2.85E-03	5.27E-02	4.79E-03	3.13E-02	4.88E-01	DC
**EV**	-4.59E-02	5.58E-02	-8.22E-01	7.36E-02	-6.23E-01	2.67E-01	DC
**TIS**	-3.79E-02	5.58E-02	-6.80E-01	7.49E-02	-5.06E-01	3.06E-01	DC
**Day:EV**	3.57E-03	3.83E-03	9.31E-01	5.03E-03	7.10E-01	2.39E-01	DC
**Day:TIS**	2.81E-03	3.83E-03	7.35E-01	5.16E-03	5.45E-01	2.93E-01	DC
**Intercept**	4.32E-01	5.78E-02	7.46E+00	6.25E-02	6.91E+00	2.43E-12	Presenting
**Day**	-1.81E-02	3.91E-03	-4.63E+00	3.97E-03	-4.55E+00	2.65E-06	Presenting
**EV**	-6.63E-02	8.18E-02	-8.10E-01	8.53E-02	-7.76E-01	2.19E-01	Presenting
**TIS**	-2.72E-01	8.18E-02	-3.33E+00	7.65E-02	-3.56E+00	1.86E-04	Presenting
**Day:EV**	1.10E-02	5.52E-03	1.99E+00	5.60E-03	1.97E+00	2.46E-02	Presenting
**Day:TIS**	1.86E-02	5.52E-03	3.37E+00	4.99E-03	3.73E+00	9.53E-05	Presenting
**Intercept**	2.25E-02	4.56E-02	4.92E-01	6.07E-03	3.70E+00	1.09E-04	Proliferating
**Day**	-5.00E-04	3.20E-03	-1.56E-01	4.24E-04	-1.18E+00	1.20E-01	Proliferating
**EV**	5.50E-02	5.08E-02	1.08E+00	1.35E-02	4.07E+00	2.31E-05	Proliferating
**TIS**	1.39E-01	5.05E-02	2.75E+00	3.23E-02	4.29E+00	8.89E-06	Proliferating

**Day:EV**	-3.78E-03	3.56E-03	-1.06E+00	8.79E-04	-4.30E+00	8.53E-06	Proliferating
**Day:TIS**	-7.85E-03	3.52E-03	-2.23E+00	2.05E-03	-3.82E+00	6.56E-05	Proliferating
**Intercept**	5.55E-03	1.11E-01	4.99E-02	9.78E-02	5.68E-02	4.77E-01	F4/80hi
**Day**	5.95E-03	7.44E-03	8.00E-01	7.33E-03	8.12E-01	2.08E-01	F4/80hi

Table S9. Generalized Estimating Equations coefficients and statistics for regressions of immune fractions in neutrophil subsets over embryonic days. Related to Figure 5F. GEE was used to fit linear models to all but immunosuppressive neutrophil subset to compare fraction of immune cells in compartments within each neutrophil subset. Immunosuppressive subset was fitted with a quadratic model. PB was used as the reference.

Coefficient	Estimate	Naive.S.E.	Naive.z	Robust.S.E.	Robust.z	P value	Cell type
**Intercept**	2.83E-03	8.35E-03	3.38E-01	4.80E-03	5.89E-01	2.78E-01	CD80
**Day**	6.48E-04	5.68E-04	1.14E+00	3.00E-04	2.16E+00	1.55E-02	CD80
**EV**	-1.93E-02	1.21E-02	-1.60E+00	6.56E-03	-2.94E+00	1.62E-03	CD80
**TIS**	-2.39E-02	1.17E-02	-2.04E+00	1.03E-02	-2.31E+00	1.05E-02	CD80
**Day:EV**	1.34E-03	8.14E-04	1.65E+00	4.27E-04	3.14E+00	8.31E-04	CD80
**Day:TIS**	1.89E-03	7.94E-04	2.39E+00	7.54E-04	2.51E+00	6.04E-03	CD80
**Intercept**	1.34E-02	3.93E-02	3.40E-01	2.19E-02	6.11E-01	2.71E-01	Conventional
**Day**	2.81E-03	2.66E-03	1.06E+00	1.52E-03	1.84E+00	3.26E-02	Conventional
**EV**	-7.82E-02	5.56E-02	-1.41E+00	3.92E-02	-2.00E+00	2.29E-02	Conventional
**TIS**	2.87E-02	5.56E-02	5.16E-01	3.16E-02	9.08E-01	1.82E-01	Conventional
**Day:EV**	7.16E-03	3.76E-03	1.91E+00	2.75E-03	2.60E+00	4.60E-03	Conventional
**Day:TIS**	7.74E-04	3.76E-03	2.06E-01	2.20E-03	3.51E-01	3.63E-01	Conventional
**Intercept**	2.77E-02	3.04E-02	9.10E-01	2.04E-02	1.36E+00	8.74E-02	Presenting
**Day**	2.09E-03	2.05E-03	1.02E+00	1.36E-03	1.54E+00	6.16E-02	Presenting
**EV**	-1.56E-01	4.30E-02	-3.62E+00	2.68E-02	-5.81E+00	3.17E-09	Presenting
**TIS**	-1.04E-01	4.30E-02	-2.42E+00	3.94E-02	-2.65E+00	4.08E-03	Presenting
**Day:EV**	1.18E-02	2.90E-03	4.07E+00	1.85E-03	6.39E+00	8.57E-11	Presenting
**Day:TIS**	9.11E-03	2.90E-03	3.14E+00	2.92E-03	3.12E+00	8.99E-04	Presenting
**Intercept**	1.00E-02	1.05E-02	9.56E-01	6.92E-03	1.45E+00	7.38E-02	Proliferating
**Day**	-1.16E-04	7.01E-04	-1.66E-01	4.39E-04	-2.65E-01	3.95E-01	Proliferating
**EV**	1.17E-02	1.49E-02	7.84E-01	8.93E-03	1.31E+00	9.56E-02	Proliferating

**TIS**	9.64E-02	1.59E-02	6.07E+00	1.53E-02	6.29E+00	1.59E-10	Proliferating
**Day:EV**	-8.77E-04	1.02E-03	-8.62E-01	5.77E-04	-1.52E+00	6.43E-02	Proliferating
**Day:TIS**	-5.30E-03	1.11E-03	-4.78E+00	1.01E-03	-5.27E+00	6.68E-08	Proliferating
**Intercept**	1.33E-02	1.25E-02	1.07E+00	7.80E-03	1.71E+00	4.37E-02	Immunosuppressive
**Day**	-4.71E-04	8.56E-04	-5.50E-01	4.97E-04	-9.47E-01	1.72E-01	Immunosuppressive
**EV**	-3.19E-03	1.86E-02	-1.72E-01	9.10E-03	-3.51E-01	3.63E-01	Immunosuppressive
**TIS**	-4.92E-01	7.36E-02	-6.69E+00	1.03E-01	-4.79E+00	8.49E-07	Immunosuppressive
**Day:EV**	3.92E-04	1.26E-03	3.11E-01	5.76E-04	6.80E-01	2.48E-01	Immunosuppressive
**Day:TIS**	8.02E-02	1.03E-02	7.80E+00	1.40E-02	5.75E+00	4.45E-09	Immunosuppressive
**Day-sq:TIS**	-2.84E-03	3.53E-04	-8.07E+00	4.59E-04	-6.20E+00	2.83E-10	Immunosuppressive

Table S10. Generalized Estimating Equations coefficients and statistics for regressions of fractions out of neutrophils in neutrophil subsets over embryonic days. Related to Figure 5G. GEE was used to fit linear models to all but immunosuppressive neutrophil subset to compare fraction out of neutrophils in compartments within each neutrophil subset. Immunosuppressive subset was fitted with a quadratic model. PB was used as the reference.

Coefficient	Estimate	Naive.S.E.	Naive.z	Robust.S.E.	Robust.z	P value	Cell type
**Intercept**	4.88E-02	3.22E-02	1.52E+00	3.50E-02	1.39E+00	8.18E-02	CD80 Neu
**Day**	2.35E-03	2.19E-03	1.07E+00	2.24E-03	1.05E+00	1.47E-01	CD80 Neu
**EV**	5.05E-04	4.65E-02	1.08E-02	4.67E-02	1.08E-02	4.96E-01	CD80 Neu
**TIS**	-9.49E-02	4.51E-02	-2.11E+00	4.08E-02	-2.33E+00	9.93E-03	CD80 Neu
**Day:EV**	-1.16E-03	3.14E-03	-3.69E-01	3.01E-03	-3.85E-01	3.50E-01	CD80 Neu
**Day:TIS**	4.34E-03	3.06E-03	1.42E+00	2.70E-03	1.61E+00	5.39E-02	CD80 Neu
**Intercept**	2.54E-01	8.77E-02	2.89E+00	9.10E-02	2.79E+00	2.64E-03	Conventional
**Day**	7.11E-03	5.92E-03	1.20E+00	5.78E-03	1.23E+00	1.10E-01	Conventional
**EV**	1.07E-01	1.24E-01	8.64E-01	1.28E-01	8.36E-01	2.02E-01	Conventional
**TIS**	3.76E-02	1.24E-01	3.03E-01	1.04E-01	3.60E-01	3.59E-01	Conventional
**Day:EV**	-4.32E-03	8.38E-03	-5.16E-01	8.25E-03	-5.24E-01	3.00E-01	Conventional
**Day:TIS**	-5.32E-03	8.38E-03	-6.36E-01	6.64E-03	-8.02E-01	2.11E-01	Conventional
**Intercept**	3.72E-01	7.89E-02	4.71E+00	1.11E-01	3.36E+00	3.90E-04	Presenting
**Day**	1.67E-03	5.33E-03	3.13E-01	7.35E-03	2.27E-01	4.10E-01	Presenting
**EV**	-3.11E-01	1.12E-01	-2.79E+00	1.31E-01	-2.38E+00	8.67E-03	Presenting
**TIS**	-4.85E-01	1.12E-01	-4.35E+00	1.26E-01	-3.84E+00	6.14E-05	Presenting
**Day:EV**	2.04E-02	7.53E-03	2.71E+00	8.65E-03	2.36E+00	9.16E-03	Presenting
**Day:TIS**	2.60E-02	7.53E-03	3.45E+00	8.49E-03	3.06E+00	1.11E-03	Presenting
**Intercept**	1.03E-01	6.89E-02	1.50E+00	5.62E-02	1.84E+00	3.30E-02	Proliferating
**Day**	-3.11E-03	4.61E-03	-6.75E-01	3.57E-03	-8.72E-01	1.92E-01	Proliferating
**EV**	3.30E-01	9.79E-02	3.37E+00	1.44E-01	2.29E+00	1.09E-02	Proliferating
**TIS**	3.72E-01	1.04E-01	3.56E+00	7.69E-02	4.84E+00	6.62E-07	Proliferating
**Day:EV**	-2.22E-02	6.69E-03	-3.32E+00	9.11E-03	-2.44E+00	7.38E-03	Proliferating
**Day:TIS**	-2.31E-02	7.29E-03	-3.16E+00	5.04E-03	-4.58E+00	2.32E-06	Proliferating
**Intercept**	1.17E-01	5.51E-02	2.12E+00	5.96E-02	1.96E+00	2.48E-02	Immunosuppressive
**Day**	-4.61E-03	3.79E-03	-1.22E+00	3.86E-03	-1.19E+00	1.17E-01	Immunosuppressive
**EV**	9.24E-02	8.21E-02	1.13E+00	7.87E-02	1.17E+00	1.20E-01	Immunosuppressive
**TIS**	-1.06E+00	3.25E-01	-3.25E+00	3.57E-01	-2.96E+00	1.54E-03	Immunosuppressive
**Day:EV**	-5.73E-03	5.56E-03	-1.03E+00	4.93E-03	-1.16E+00	1.23E-01	Immunosuppressive
**Day:TIS**	1.84E-01	4.54E-02	4.04E+00	4.86E-02	3.78E+00	7.82E-05	Immunosuppressive
**Day-sq:TIS**	-6.67E-03	1.56E-03	-4.28E+00	1.61E-03	-4.15E+00	1.67E-05	Immunosuppressive

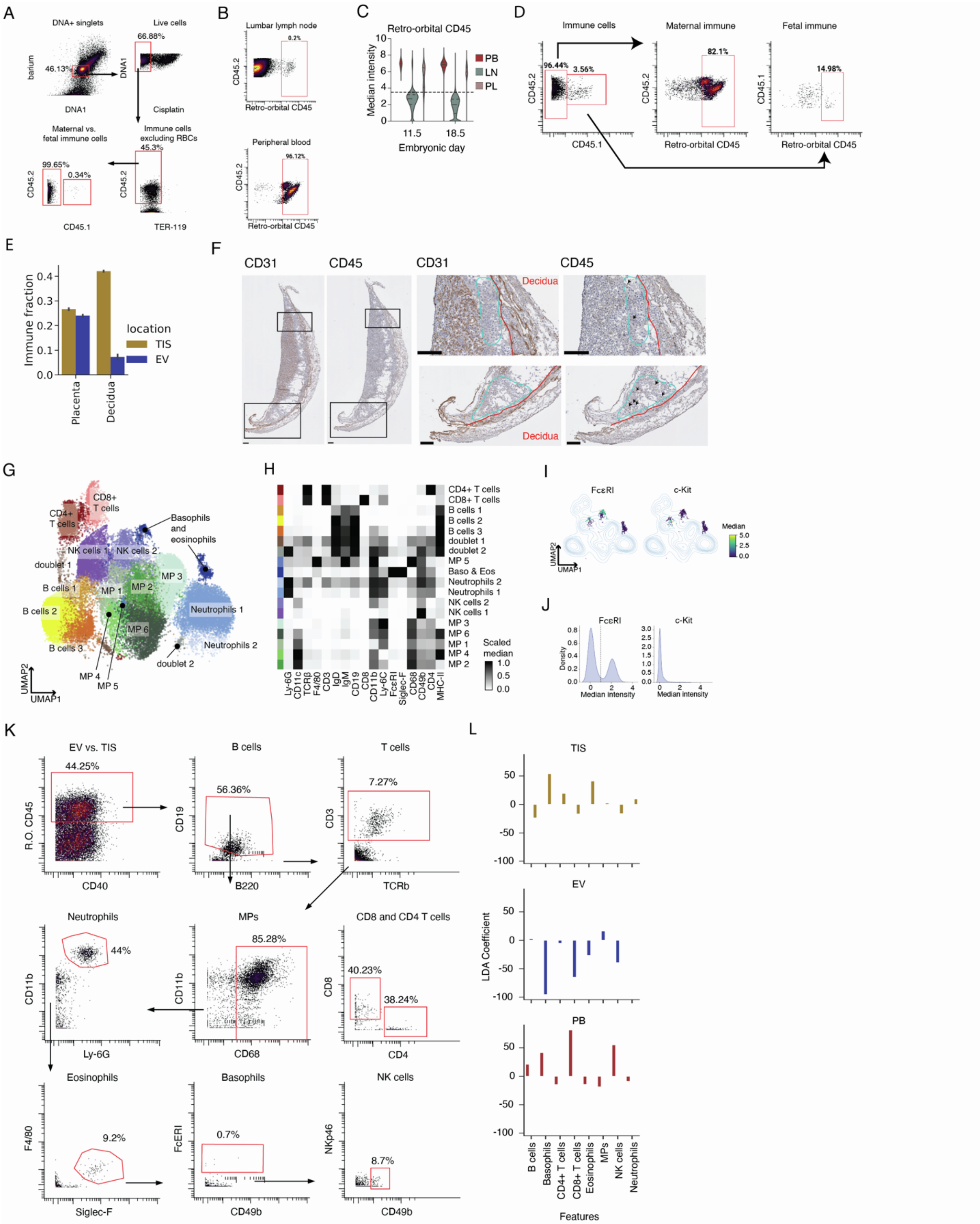

Figure S1. Maternal immune clusters identified by Leiden, determination of endovascular and tissue compartments, and gating strategy. Related to Figure 1. (A) Mass cytometry gating strategy to isolate maternal and fetal immune cells. (B) Representative biaxial plots of immune cells in paired lumbar lymph node and peripheral blood in mice retro-orbitally injected

with anti-CD45. Gated populations of immune cells labeled by retro-orbital anti-CD45. (C) Arcsinh transformed single cell median expression of retro-orbitally injected anti-CD45 in matched peripheral blood (PB), lumbar lymph nodes (LN), and placenta (PL). Plot includes the threshold set to distinguish EV vs. TIS compartments in PL. (D) Representative gating scheme of maternal vs. fetal immune cells in E18.5 maternal-fetal interface sample. Maternal or fetal immune cells labeled by retro-orbital anti-CD45 are gated in subsequent plots. (E) Fraction of maternal immune cells in TIS or EV space in mechanically dissociated placenta from decidua. E12.5, n = 2. (F) IHC of mouse maternal-fetal interface at E13.5. Serial sections were stained with CD31 or CD45. Red line on zoomed in sections indicate the decidua. Cyan region indicates absence of vasculature within the placenta. Arrows point to maternal immune cells in the placenta. Scale bar is 250 um. (G) Leiden clusters overlaid on UMAP graph of maternal immune cells. Leiden clusters were named based on their phenotypic marker expression. (H) Scaled median expression of protein markers used to generate UMAP graph and Leiden clusters are shown across all Leiden clusters identified. (I) UMAP indicating arcsinh transformed cellular median intensity of FcεRI and c-Kit in the Leiden cluster identified as “Basophils and Eosinophils”. (J) Kernel density plots of arcsinh transformed FcεRI and c-Kit medians in the Leiden cluster identified as “Basophils and Eosinophils”. FcεRI plot includes the threshold set to distinguish basophils from eosinophils. (K) Gating strategy to define EV vs. TIS in placenta E12.5 sample, along with the maternal immune cell types identified. (L) Linear discriminant analysis (LDA) coefficients indicating the weight each feature (cell type) was given to identify each class (compartments TIS, EV, and PB).

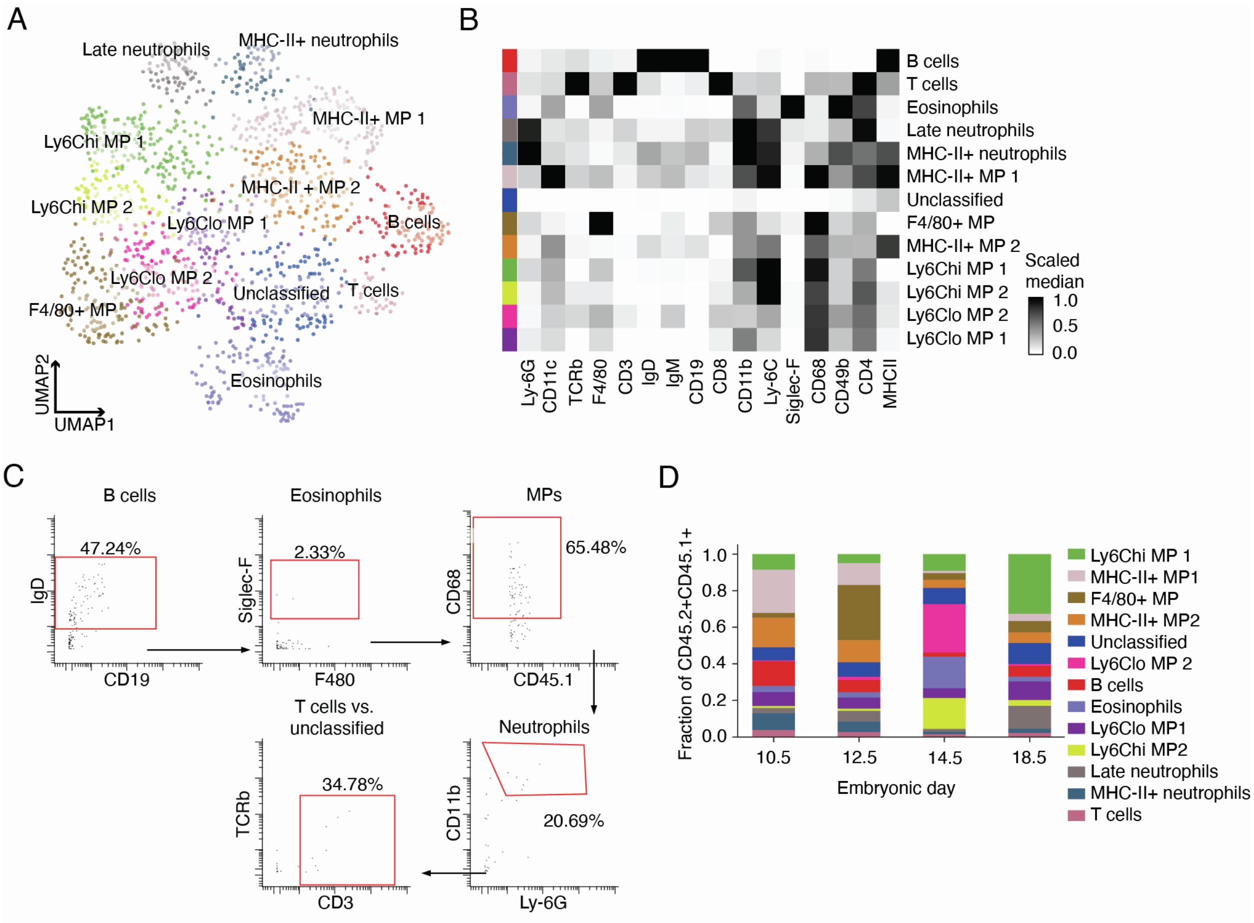

Figure S2. Fetal immune clusters identified by Leiden and their gating strategy. Related to Figure 2. (A) Leiden clusters overlaid on UMAP graph of fetal immune cells. Leiden clusters were named based on their phenotypic marker expression. (B) Scaled median expression of protein markers used to generate UMAP graph. Proteins are shown across all Leiden clusters identified. (C) Gating strategy to identify fetal immune cell types. (D) Fraction of fetal immune cells at E10.5, 12.5, 14.5, and 18.5 across Leiden clusters.

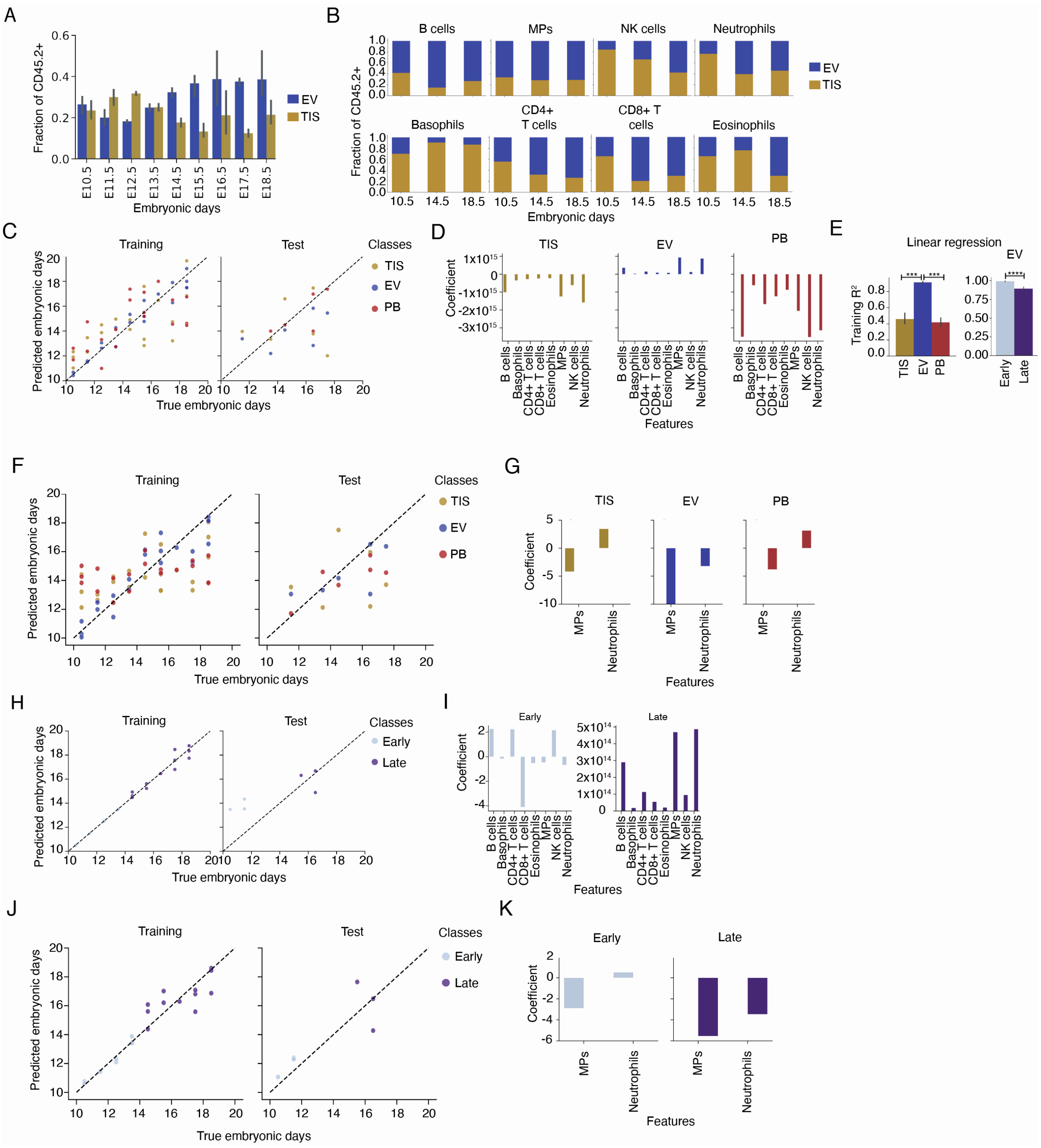

Figure S3. Frequencies of maternal immune cell subsets show predictive dynamics throughout gestation. Related to Figure 3. (A) Fraction of maternal immune cells in the maternal-fetal-interface TIS and EV compartments across embryonic days. (B) Fraction of maternal immune cell subsets in the maternal-fetal-interface TIS and EV compartments across embryonic days 10.5, 14.5, and 18.5. (C) Linear regression results by maternal compartment (classes) based on maternal immune cell fractions out of all immune cells in the given compartment (features). The training set is made up of previously seen data, and the test set is made up of data that has not been seen by the linear regression algorithm. (D) Linear regression coefficients for features, in this case cell type, within the specified class, or maternal compartment. (E) Training accuracy as determined by R^2 of linear regression across each

compartment and EV period based on cell fractions across embryonic days. ***p ≤ 0.001, ****p ≤ 0.0001 (one-way ANOVA for comparing compartments, unpaired t test for early and late stages). (F) Linear regression results by maternal compartment (classes) based on maternal immune cell fractions of mononuclear phagocytes and neutrophils in the given compartment (features). (G) Linear regression coefficients for features, in this case cell type, within the specified class, or maternal compartment. (H) Cell fractions of EV compartment were split into early (E10.5 - 13.5) and late (E14.5 – 18.5) and linear regression was run independently for the early and late periods. Linear regression results on the training and test sets based on maternal immune cell fractions (features). (I) Linear regression coefficients for features, in this case cell type, within the specified class, or EV embryonic period. (J) Cell fractions of mononuclear phagocytes and neutrophils EV compartment were split into early and late and linear regression was run independently for the early and late periods. (K) Linear regression coefficients for features, in this case cell type, within the specified class, or EV embryonic period.

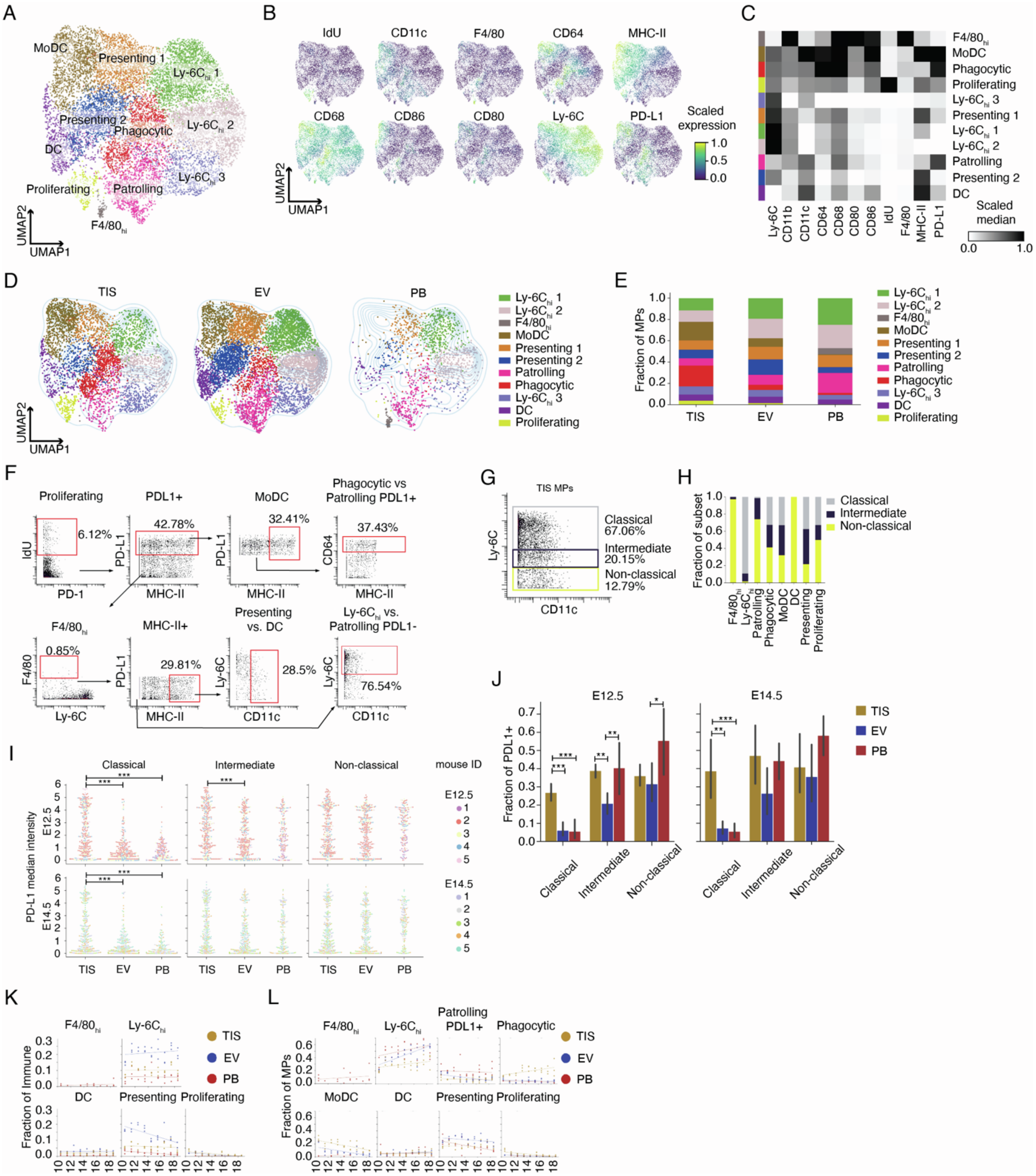

A
B
C
D
E
F
G
H
I
J
K
L
MoDC
Presenting 1
Presenting 2
Phagocytic
Proliferating
Patrolling
DC
F4/80hi
Scaled expression
Scaled median
TIS
EV
PB
Fraction of MPs
TIS MPs
Classical 67.06%
Intermediate 20.15%
Non-classical 12.79%
Fraction of subset
PD-L1 median intensity
mouse ID
E12.5
E14.5
Fraction of PDL1+
Fraction of Immune

Figure S4. Maternal mononuclear phagocyte clusters identified by Leiden, their gating strategy, and canonical monocyte subset phenotypes. Related to Figure 4. (A) Leiden clusters overlaid on composite UMAP graph of maternal mononuclear phagocytes (MPs) in placenta and peripheral blood across E10.5 to 18.5. Leiden clusters were named based on their phenotypic marker expression. (B) Scaled cellular median intensity of MP phenotypic markers. (C) Scaled median expression of protein markers are shown across all Leiden clusters identified. (D) UMAP plots showing distribution of MP clusters identified by Leiden across compartments. (E) Fraction of MP clusters out of total MPs in each compartment. Embryonic days were aggregated. (F) Gating strategy to identify MP subsets. (G) Gating strategy to identify canonical MP subsets, classical, intermediate, and non-classical. (H) Fraction of canonical MP classification for each MP subset. (I) Single cell arcsinh median intensity of PD-L1 in canonical MP subsets across compartments and at E12.5 and 14.5. To clearly show single cell distribution, samples were capped at 30 cells per mouse. (J) PD-L1 positive fraction of each canonical MP subsets across compartments and at E12.5 and 14.5. For (I) and (J), significance is shown as $*p \leq 0.05$, $**p \leq 0.01$, $***p \leq 0.001$ (one-way ANOVA per cell type). (K) Fraction of PD-L1 negative MP subsets out of maternal immune cells were fitted with GEE using a linear model comparing TIS, EV, and maternal PB from E10.5 to 18.5. (L) GEE was used to fit a linear model to cell fractions of MP subsets out of all MPs comparing TIS, EV, and PB from E10.5 to 18.5.

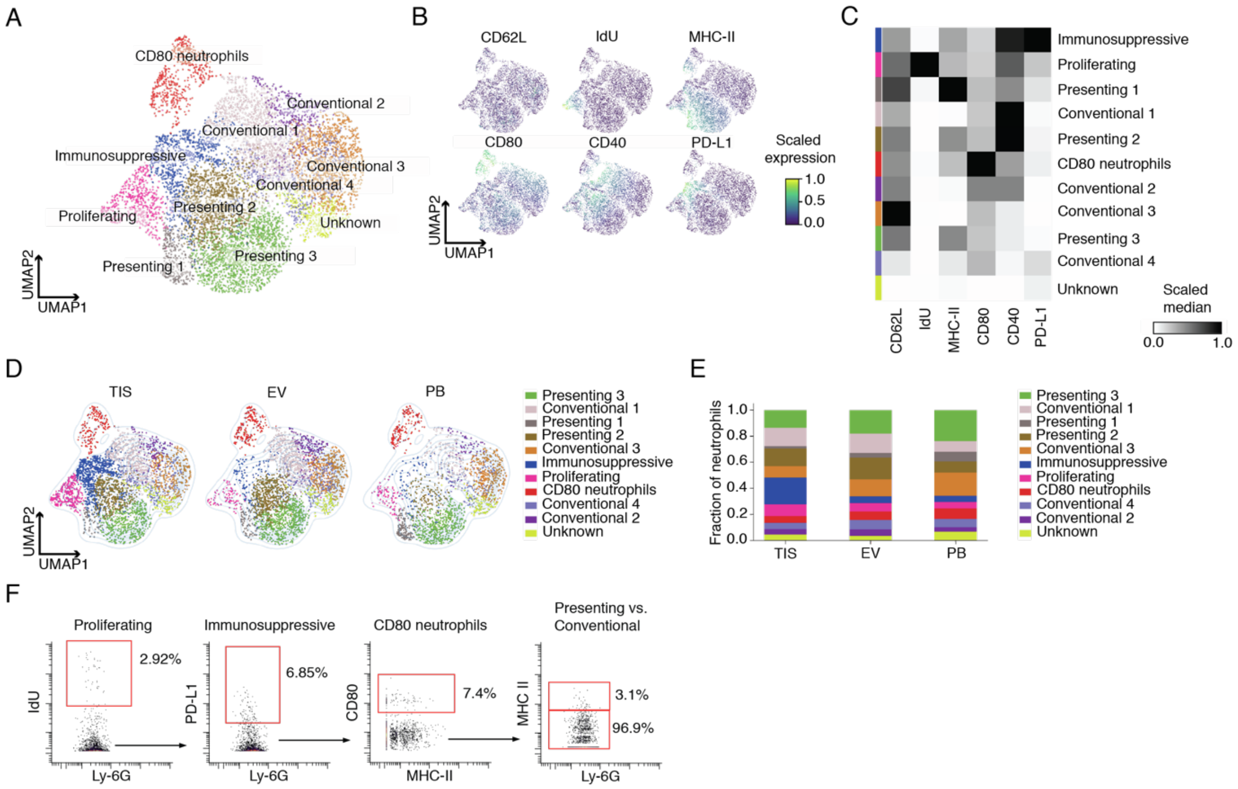

Figure S5. **Maternal neutrophil clusters identified by Leiden and their gating strategy.** Related to Figure 5. (A) Leiden clusters overlaid on composite UMAP graph of maternal neutrophils in placenta and peripheral blood across E10.5 to 18.5. Leiden clusters were named based on their phenotypic marker expression. (B) Scaled cellular median intensity of neutrophil phenotypic markers. (C) Scaled median expression of protein markers are shown across all Leiden clusters identified. (D) UMAP plots showing distribution of neutrophil clusters identified by Leiden across compartments. (E) Fraction of neutrophil clusters out of total neutrophils in each compartment. Embryonic days were aggregated. (F) Gating strategy to identify neutrophil subsets.

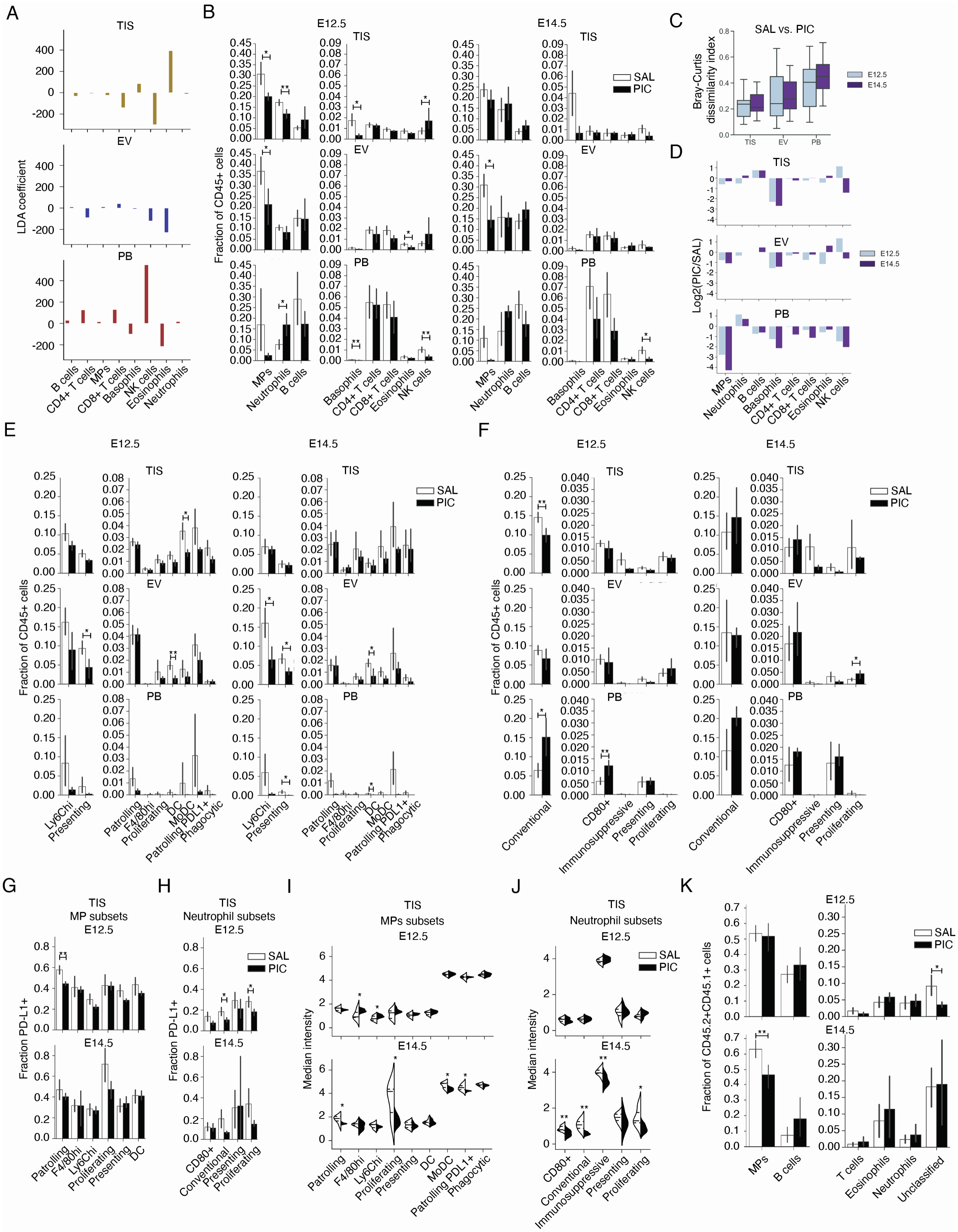

Figure S6. **Maternal and fetal immune responses to maternally, systemically administered Poly(I:C) at mid gestation.** Related to Figure 6. (A) Linear discriminant analysis (LDA) coefficients for cell type (features), for each compartment (class). LDA was trained on baseline

data comprised of E12.5 and 14.5. (B) For E12.5 and 14.5, maternal immune cell type fractions across compartments when challenged with saline (SAL) or Poly(I:C) (PIC). (C) Bray-Curtis dissimilarity between PIC and SAL treated animals for each compartment across E12.5 and E14.5. (D) Log2 ratio between PIC and SAL across compartment and cell type comparing E12.5 and E14.5. (E) For E12.5 and 14.5, fractions of maternal MP subsets across compartments when challenged with SAL or PIC. (F) For E12.5 and 14.5, fractions of maternal neutrophil subsets across compartments when challenged with SAL or PIC. (G) PD-L1 positive fractions of PD-L1 low maternal MP subsets at E12.5 and 14.5, in TIS compartment when challenged with SAL or PIC. (H) PD-L1 positive fractions of PD-L1 low maternal neutrophil subsets at E12.5 and 14.5, in TIS compartment when challenged with SAL or PIC. (I) Arcsinh transformed median intensity of PD-L1 for MP subsets in TIS at E12.5 and 14.5 upon SAL or PIC challenge. (J) Median intensity of PD-L1 for neutrophil subsets in TIS at E12.5 and 14.5 upon SAL or PIC challenge. (K) For E12.5 and 14.5, fetal immune cell fraction was compared for immune challenge with SAL or PIC. For (A) and (E) through (K), significance is shown as $*p \leq 0.05$, $**p \leq 0.01$, $***p \leq 0.001$ (unpaired t test).
